# The geography and timing of genetic divergence in the lizard *Phrynocephalus theobaldi* on the Qinghai-Tibetan plateau

**DOI:** 10.1038/s41598-017-02674-4

**Published:** 2017-05-23

**Authors:** Yuanting Jin, Naifa Liu, Richard P. Brown

**Affiliations:** 1 College of Life Sciences, China Jiliang University, Hangzhou, 730000 P. R. China; 2 School of Life Sciences, Lanzhou University, Lanzhou, 730000 P. R. China; 3 School of Natural Sciences and Psychology, Liverpool John Moores University, Liverpool, UK

## Abstract

The Qinghai-Tibetan Plateau (QTP) represents one of the earth’s most significant physical features and there is increasing interest in the historical generation of biodiversity within this region. We hypothesized that there should be clear geographically coherent genetic structuring within one of the world’s highest altitude lizards, *Phrynocephalus theobaldi*, due to considerable historical population fragmentation in this environment. This was tested using a major mitochondrial DNA (mtDNA) survey and sequencing of two nuclear markers (*AME* and *RAG-1*) from *P. theobaldi*, from across the southern QTP. A Bayesian method (BPEC) was used to detect four geographically structured mtDNA clusters. A Bayesian phylogenetic tree, together with associated dating analyses, supported four corresponding evolutionary lineages with a timing of 3.74–7.03 Ma for the most basal *P. theobaldi* split and Pliocene splits of 2.97–5.79 Ma and 2.40–5.39 Ma in the two daughter lineages. Himalayan uplift and changes in the Jilong basin may have contributed to these divergences, but uplift of the Gangdese mountains is rejected due to its timing. The nuclear markers appeared to be sorted between the four mtDNA groups, and species delimitation analyses supported the four phylogeographical groups as candidate species. The study contributes to our understanding of biodiversity on the QTP.

## Introduction

The generation of biodiversity on the Qinghai-Tibetan Plateau (QTP) has become a focus for evolutionary research^1–9^. Miocene events on the plateau appear to have had an impact at the intrageneric level or higher. For example, divergence of viviparous from oviparous lineages of *Phrynocephalus* lizards is thought to have occurred at approximately this time^10^ as did divergence of *Thermophis* snake species^11^. Many other studies have documented more recent biodiversity-generating mechanisms up to and including the last major glaciation, which appear to have impacted population structure within several species, including snow finches^3^ and pikas^12^. Between these ancient and more recent effects, intermediate divergence that corresponds to intra- or low-level interspecific divergence has also been documented and linked to regional physical effects within the plateau^9^.

Many of these studies have associated QTP uplift with recent diversification, but this has been questioned based on the premise that the plateau reached its present height at least 15 Ma ago and probably much earlier, during the Eocene^13^. In other words, if reported timings of divergence are correct, then plateau uplift may not be the correct explanation. While more recent geological studies support the hypothesis that the centre of the QTP reached its present elevation quite early, there is also plenty of evidence of more recent uplift in other parts of the Tibetan Plateau^14^. For example, there is evidence that the Himalayas and the Southern edge of the QTP did not reach their current height until the late Miocene^14, 15^. Robust analyses of genetic diversity that examine geographical and temporal aspects of divergence within lower taxonomic units, particularly with regard to specific geological structures, could help to shed more light on the potential role of uplift on diversification on the QTP.

To date there have been relatively few animal diversity studies that have analysed the southern QTP but see^16, 17^. Here we investigate a very interesting lizard which is little-known outside of China, *Phrynocephalus theobaldi*. This species inhabits elevations of 3600–5100 m and is therefore one of the highest altitude reptiles on earth^10, 18^. It almost certainly lives close to the upper limit of a lizard’s habitable environment^10, 18^. *Phrynocephalus theobaldi* has an elongated distribution, extending over 1000 km due to its occupation of narrow mountain valleys, which cover the western and southern portions of the Xizang area of the QTP, including the Ngari region of western Tibet, the Brahmaputra River valley, and the Xizang Southern Valley, between the north side of the Himalaya mountains and the south side of the Gangdese-Nyainqentanglha mountains^18^.

Two subspecies are currently recognized on the basis of morphological differences^19^: *P. theobaldi theobaldi* (higher regions of Ngari in the western Xizang area) and *P. theobaldi orientalis* (middle regions of Brahmaputra River valley and the lower regions of the Xizang Southern Valley), which suggests possible genetic differences. We hypothesized that if population fragmentation was caused by physical events (such as orogenic uplift) then major geographical structuring would correspond to the relevant geological features, as would timings of intraspecific divergence. We tested this hypothesis by means of a major mtDNA survey, identification of phylogeographic clusters, divergence time dating of these clusters, and finally, analyses of nuclear DNA sequences to investigate concordance of patterns between different loci, which also provides insights into whether clusters might represent valid species (although this study does not aim to describe new taxonomic units).

## Results

### MtDNA haplotypes and sequence characteristics

**Figure 1:**
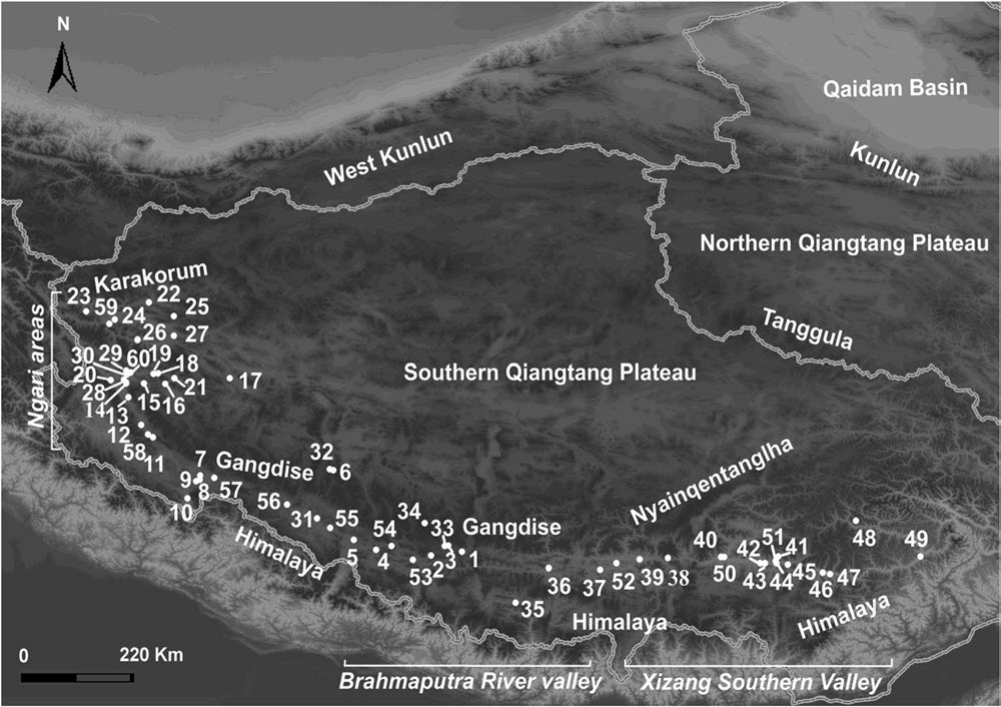
The *Phrynocephalus theobaldi* sample sites, numbered 1–60, on the Southern Tibetan Plateau, China, and surrounding region, from which the 345 specimens were obtained (map data were provided by the Scientific Data Centre of the Chinese Academy of Sciences and the final map produced by the authors using the software ArcGis 9.1).

Sequencing of PCR products for *P. theobaldi* provided 578 bp of mtDNA, with 168 variable sites. Eighty three mtDNA haplotypes were detected. Split by traditional subspecies, there were 39 haplotypes in 172 *P. t. theobaldi* and 44 haplotypes in 182 *P. t. orientalis*. Detailed information on mtDNA haplotype and nuclear genotype sequences, and their correspondence with individuals and with the sample sites shown in Fig. 1 are provided in the Supplementary information (S1 and S2).

The χ^2^ test of base compositional bias for the entire mtDNA alignment including the outgroup species (*X* ^2^_[447]_ = 94.26, *P* = 1.00) ruled out any potentially adverse effects on phylogenetic inference^20^. Nucleotide composition showed an anti-G bias (G = 8.2%). A total of 200 polymorphic sites (34.6%) were recorded across all species, of which 134 (23.18%) were parsimony-informative and provided a transition: transversion ratio of 6.47:1.

### Networks, phylogenetic and phylogeographic analyses

**Figure 2:**
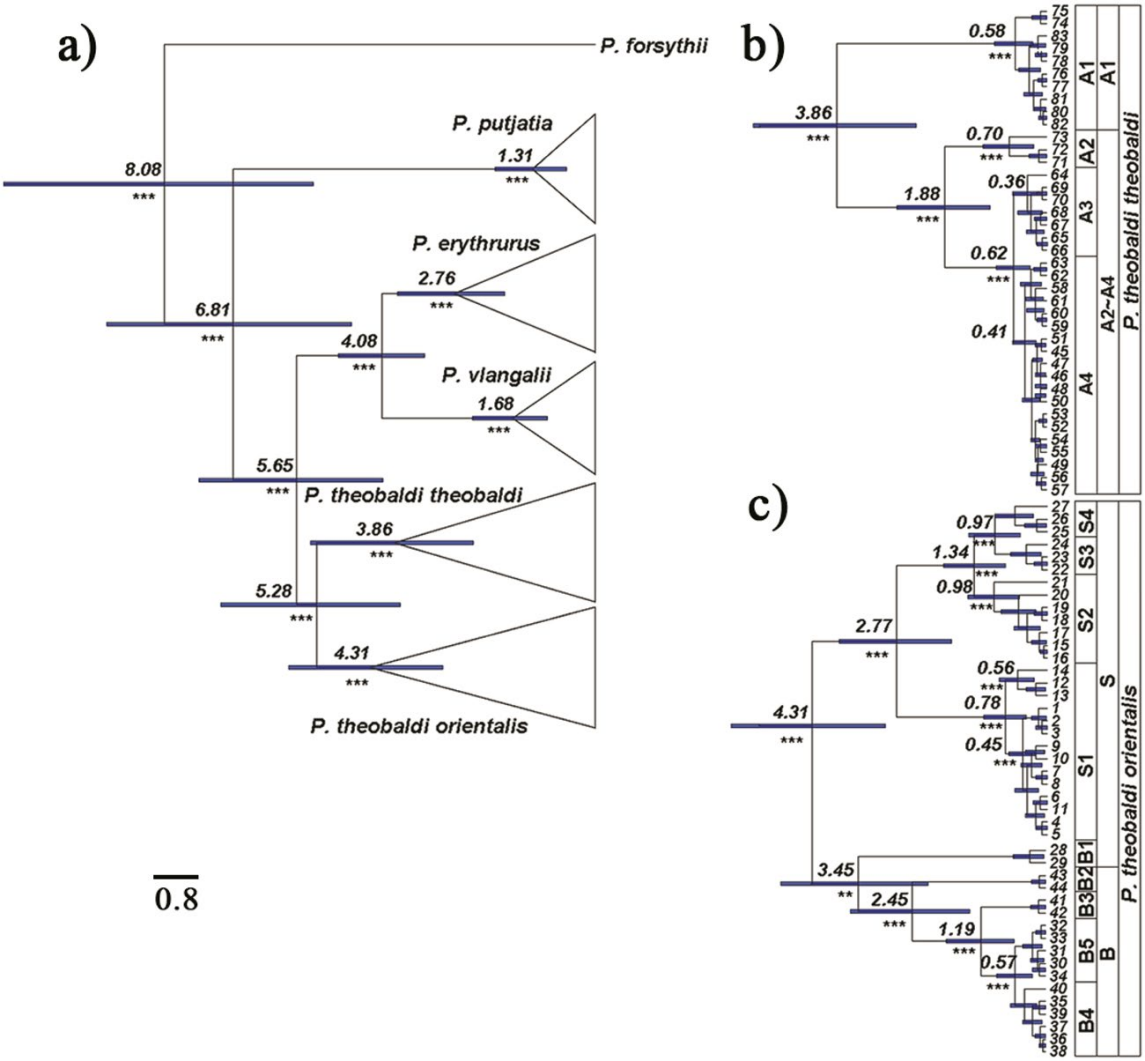
The Bayesian tree on the left (Fig. 2a) shows divergence times across related *Phrynocephalus* species (*P. forsythii*, *P. vlangalii*, *P. erythrurus*, *P. putjatia* and *P. theobaldi*), as obtained from Bayesian dating analyses. Higher resolution detail of relationships and divergence times among numbered haplotypes within the two *P. theobaldi* clades of interest are shown on the right (Fig. 2b and c). Values on nodes are posterior mean divergence times, bars represent the 95% HPDs for these times. Time-calibrated nodes are denoted by #. Posterior node support values for nodes that were not constrained to be monophyletic are given as asterisks: *>0.75, **>0.90, ***>0.95.

The Bayesian mtDNA tree revealed two well supported major lineages in *P. theobaldi* (Fig. 2a; site numbers, site locations and haplotype compositions are given in Supplementary Information S1). The lineage we described as clade A corresponded to the currently recognized subspecies *P. t. theobaldi* and comprised four subclades (A1-A4) from the Ngari region. The sister lineages we describe as clades B and S correspond to the other recognized subspecies, *P. t. orientalis*. Clade B was mainly distributed around the Brahmaputra River valley and clade S was found in the Xizang Southern Valley (Fig. 2b). Clade B was divided into five subclades (B1-B5) and clade S was split into four subclades (S1-S4). Geographic distributions of these major lineages/clades were non-overlapping with the exception of clade B1 which was found with subclades S2 and S4 at two sites (Fig. 2b). Also, within the S clade, each of S2–S3, S2–S4 and S3–S4 overlapped at one site.

**Figure 3:**
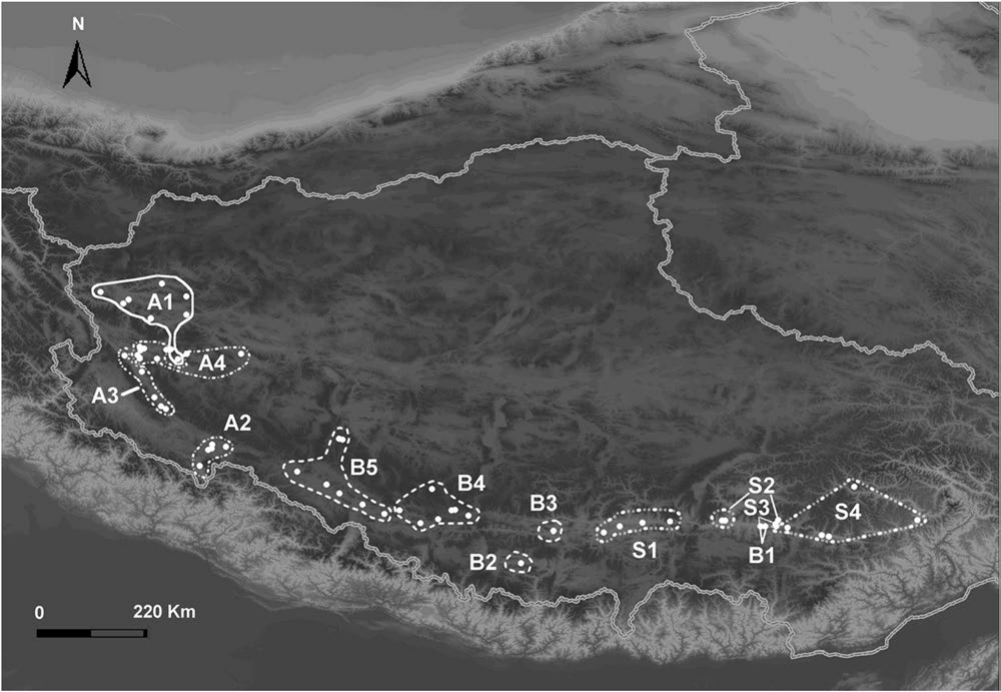
The geographical distributions of the main *P. theobaldi* mtDNA groups within the three main clades (A, B and S) that were identified by a Bayesian analysis of all mtDNA haplotypes (map data were provided by the Scientific Data Centre of the Chinese Academy of Sciences and the final map produced by the authors using the software ArcGis 9.1).

Bayesian estimates of mtDNA divergence times are given for the major nodes in the tree (Fig. 2a: phylogenetic detail for the other taxa in the tree are shown in Supplementary Information S5). The first mtDNA divergence within *P. theobaldi* is dated at 5.28 (95% highest posterior density [HPD]: 3.74–7.03) million years (Ma). The timing of divergences within clade A were also quite old: 3.86 (95% HPD: 2.40–5.39) Ma for the splitting of subclade A1 from A2-A4, down to 0.62 (95% HPD: 0.31–0.93) Ma for divergence of the most recent subclades (A3 and A4) (Fig. 2b). The oldest posterior mean divergence time within clades B and S was 4.31 (95% HPD: 2.97–5.79) Ma for the split between these two clades, and the most recent was 0.57 (95% HPD: 0.25–0.90) Ma for the divergence between the subclades B4 and B5 (Fig. 2c). The geographical distributions of these clades are given in Fig. 3. Dates from the maximum likelihood (ML) dating analysis were generally very similar to posterior means from the BEAST analyses, suggesting that the prior on divergence times had little influence on these dates which supports the robustness of our divergence time estimates. For example, the earliest ML divergence estimate within *P. theobaldi* was 5.29 Ma, virtually identical to the posterior mean under BEAST, and the main splits within clades A and B/S were 3.97 Ma and 4.34 Ma, respectively (again, very similar to BEAST estimates).

The BPEC parsimony network is given in S3 Supplementary information (note that haplotype 36 was grouped with haplotype 38 in this analysis because of the way missing site information is treated by the program). The network contains one loop that leads to ambiguous connections between haplotypes. The haplotype groupings in the parsimony tree (not shown) correspond to the major lineages detected by BEAST, in terms of the four main subclades within clade A and nine divergent subclades within clades B and S.

**Figure 4:**
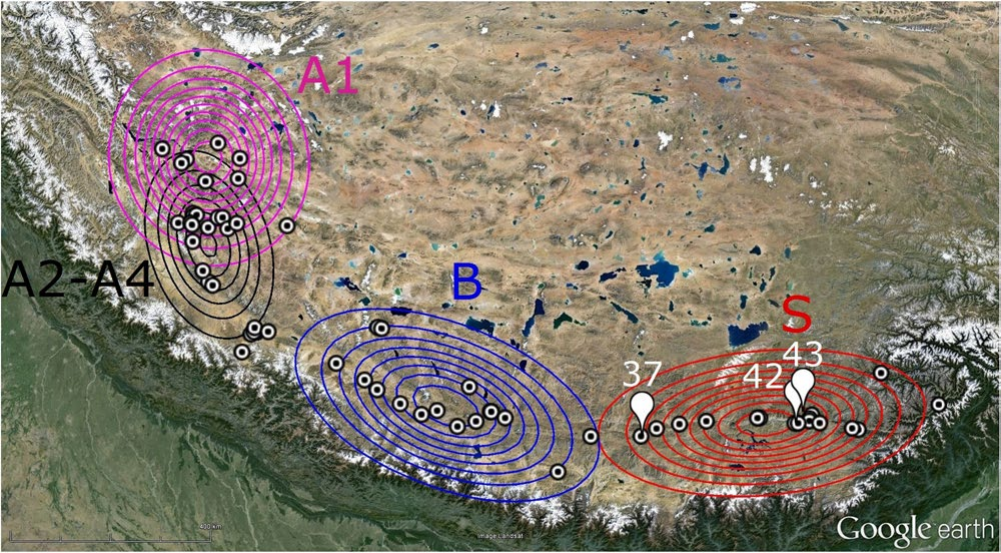
Bivariate normal contour plots showing the posterior distributions of the four phylogeographic clusters identified in *P. theobaldi* (A1, A2-A4, B and S) identified by BPEC, evaluated at the posterior means. Sample sites are given as circles, with the three inferred ancestral sites (37, 42, 43) also marked with a callout symbol. The background satellite map is from Google Earth (attribution: Image Landsat).

BPEC clustering assigned haplotypes to one of four phylogeographic clusters with generally high posterior probabilities (0.62–0.98) (S3 Supplementary information). However, haplotypes 41 and 42 both had similarly low probabilities for two alternative clusters (posterior probabilities of ~0.4), and so could not be unambiguously assigned to a single cluster. With the exception of these haplotypes and haplotypes 28 and 29 (which were allocated by BPEC to a cluster that contained individuals of clade S), the haplotype composition of each of the four clusters corresponded exactly with each of the four most divergent BEAST clades/subclades. Hence, for clarity, the BPEC clusters are given the same names as the clades identified by the phylogenetic analysis, i.e., cluster A1, cluster A2-A4 cluster B and cluster S. The posteriors on two of the clusters, A1 and A2-A4, show strong geographical overlap, suggesting (mtDNA) migration while posteriors on the other two clusters show no overlap between themselves or with A1 and A2-A4 (Fig. 4). Sites with the highest posterior probabilities as ancestral population locations were sites 37, 42, and site 43, which are all within the Southern Tibetan Valley and correspond to clade S (Fig. 4).

### Nuclear DNA and species delimitation

We obtained 901 bp of *RAG-1* (18 variable sites) and 383 bp of *AME* sequence (8 variable sites) from 103 *P. theobaldi*. Median-joining networks showing relationships among nuclear sequences are provided in the S4 Supplementary information. Sequenced individuals were assigned to one of four proposed species, based on the BPEC phylogeographic clusters. Sample sizes for each cluster were: 14 (corresponding to clade A1 in BEAST), 43 (clades A2-A4), 23 (clade B), 23 (clade C). Two specimens with mtDNA haplotypes 41 and 42 were assigned to the species that contained individuals with mtDNA clade B based on the Bayesian tree (due to the ambiguity of the BPEC assignment: see above), while nuclear loci were not sequenced from individuals with haplotypes 28 and 29.

There was very strong statistical support for species delimitation of the four BPEC clusters, when the mtDNA tree was used as a guide tree (indicating high levels of concordance between markers). The posterior probability for the full delimitation was 1.00 for all (replicated) analyses and this was insensitive to different specifications of the priors on population sizes and divergence times. The same levels of statistical support were also obtained for analyses that integrated over all species histories (all species were supported by a posterior probability of 1.00).

## Discussion

Here, deep and relatively ancient geographically structured divergence was detected within *P. theobaldi*, which was concordant between nuclear and mtDNA markers. The timing of the first speciation event in *P. theobaldi* (approximately 5.3 Ma) fell within a likely period of tectonic uplift of the Himalaya region at 3.6–7.4 Ma^21^, with 5 Ma being reported as one of the most intense Neogene periods of regional deformation^22^. The descendant lineages are found on different mountainous basins and they may have become isolated at this time. The formation of many of these mountainous basins on the QTP could have caused significant climatic change at aprroximately 5.7 Ma^23^. However, there is considerable debate about these QTP events, with some authors supporting a decrease in elevation over this period^24^. The available physical data are debatable and so do not allow us to demonstrate a temporal link with uplift, climate change or other events. Nevertheless, the basal *P. theobaldi* split is interesting because it is one of the oldest dates obtained for intraspecific divergence on the plateau. This may explain why we found support for delimitation of both of the descendant lineages into separate species: protracted periods of little/no introgression would be expected to lead to greater lineage sorting of all markers. To emphasize the depth of this split, it should be pointed out that phylogenetic studies of endemic QTP species including several birds^5, 25, 26^, frogs^27^ and mammals^28, 29^, have revealed more recent interspecific splits, among closely related species. Also, a study of catfish detected intergeneric divergence times similar to those that we describe here^30^.

One of the descendant lineages (clade A) corresponds to the morphological subspecies *P. t. theobaldi*. This clade also contains ancient divergence (estimated here at 3.8 Ma) that has given rise to two sublineages (A1 and A2-A4). Species delimitation of A1 and A2-A4 is also supported. One of these candidate species (A1) is isolated in mountainous basins or plains between the northern margin of the northwestern extensions of the major Gangdese mountains and the southern margin of the Karakoram mountains in Rutog County (Ngari region). A1 is geographically separated from its candidate sister species (A2-A4) by the Gangdese mountains (Figs 1 and 3). Current evidence suggests that these mountains reached their present heights some 10–20 Ma^31^, so while they appear to form a barrier, their uplift may not be the cause of fragmentation that has led to the generation of two candidate species.

Similarly, the other main clade comprises two subclades (B and S) that seem to have diverged slightly before divergence in clade A, i.e., 4.3 Ma. Together, B and S correspond to the subspecies *P. t. orientalis*. Species delimitation of B and S is also supported, suggesting generally concordant divergence of nuclear and mtDNA markers. Clade S is distributed in the Xizang Southern Valley between the Nyenchentanglha mountains and the eastern extent of the Himalayan mountains while lineage B is found in the Brahmaputra River valley between the Gangdese and Himalayan mountains. The ancient timing of this proposed speciation (~4.3 Ma) also coincides with significant climatic changes (2.5~5.7 Ma)^32^ induced by tectonic uplift of the Himalayas beginning 7.0 Ma^14, 32^. The Jilong basin, is located between clades B and S and contained a large paleolake that is thought to have reached its maximum size 5.9–3.6 Ma^33^. This period coincides with or just postdates major changes in the horse paleofauna of the region^33^. Changes in the size of another QTP paleolake (in the Qaidam basin) also seem to be closely associated with evolution within a congeneric species from the QTP: *P. vlangalii* ^8^. Hence, although there is some uncertainty and debate over the timings of geological events in the region, clade distributions and divergences show some links to specific physical events.

In this study we applied the Bayesian phylogeographic clustering algorithm in BPEC which is aimed at examining dispersal rather than vicariance^34^. Historical fragmentation could lead to a distribution in which several haplotypes are shared by say two population clusters, but their descendants are all unique to one cluster. Manolopoulou *et al*.^34^ showed how the current clustering approach in BPEC does not directly account for this scenario (although this could be remedied by modifications to the algorithm). An additional problem is that the assignment of individuals to clusters can sometimes be unclear. It might be useful to establish threshold criteria, based on posterior probabilities, above which the assignment is accepted. Wider use of BPEC would help determine whether or not this represents a frequent problem. Here, the posterior probabilities led to quite unequivocal assignments in all but two cases, which did not pose a significant problem. Nonetheless, we deemed several relatively low posterior probabilities of 0.63–0.65 to be unambiguous because they were greater than 0.5 and approximately twice the magnitude (or greater) of posterior probabilities for assignments to alternative clusters. Future investigation of what constitutes unambiguous assignment would be useful.

Our species delimitation analyses were based on an mtDNA guide tree. While we essentially used this to examine whether nuclear and mtDNA markers showed the same patterns, it also allowed us to propose phylogeographic groups as candidate species. Geographic discordance of nuclear-mtDNA may provide potential difficulties although it should lead to lower posterior probabilities for species delimitations rather than false positives. Another possibility is that high mtDNA substitution rates led to far more phylogeographic clusters than would be detected by nuclear loci. Again, these clusters would not be statistically delimited due to low information content of the latter and so we should not expect false species detection.

In summary, our approach has allowed detection of four phylogeographical groupings within this species across its distribution in the southern Tibetan plateau. The two most eastern and two most western clusters correspond to respective previously-described morphological subspecies. The four clusters largely correspond to distinct mountain basins and valleys at different elevations and are supported by both nuclear and mtDNA. All of the clusters seem to have originated during early Pliocene or late Miocene events on the QTP and show some evidence of links to specific physical events. The age of the intraspecific divergence has left strong genetic signatures on different markers that allows statistical delimitation of genetic groups into four separate species.

## Methods

### Specimens and sample sites

Specimens were sampled from 60 sites during expeditions to the QTP in 2007, 2010 and 2011 (Fig. 1, S1 Supplementary information), covering the known range of the *P. theobaldi* group^35^. Tail tips or liver samples were preserved in 100% ethanol after capture. Voucher specimens are held in the Department of Biology, College of Life Sciences, China Jiliang University, Hangzhou.

Fieldwork and tissue sampling authorization was provided by the Tibet Autonomous Region Forestry Bureau (TARFB). All experimental protocols were performed in accordance with instruction guidelines from the China Council on Animal Care and approved by the guidelines of the Ethics Committee of Animal Experiments at China Jiliang University.

### Laboratory procedures

Total DNA was extracted using standard phenol-chloroform techniques^36^. DNA sequences were obtained from the mitochondrial genome and two nuclear loci using Sanger sequencing of PCR products: (i) a 602–606 bp mtDNA fragment that included part of the ND2 gene and adjacent tRNA^Trp^ (complete) and tRNA^Ala^ (partial) genes (primer pair ND2 L5002 and Ala H5617b, described in Jin *et al*.^8^, (ii) a 987 bp of the nuclear recombination activating gene 1 (*RAG-1*) using primers JRAG1f.1 and JRAG1r.13^37^ and also a new primer-pair designed for this study: RAG17s (50-TCARGCAAACCTTCAGAACC-30) and RAG17a (50-CAGGAACARAGTTAGGCACA-30) and (iii) 392 bp of the nuclear Amelogenin (AME) gene using LAM2N and HAM primers^38^. All PCR products were commercially sequenced.

### Phylogenetic analyses of mtDNA

Bayesian analyses were performed in BEAST (v1.8.2)^39^ to infer the mtDNA tree and investigate lineage divergence times. Previously published mtDNA haplotype sequences from the related species, *P. putjatia*, *P. vlangalii* and *P. erythrurus* were also incorporated in the analyses to enable the use of time calibrations. These were: *P. erythrurus* (EF375637-41)*, P. vlangalii* (EP375642-81)*, P. putjatia* (EF375623-36) and *P. forsythii* (EF375684). We applied four monophyly constraints to the internal nodes of the tree following the findings of a previous cross-species study^10^. The nodes were: i) the second most basal node which incorporated all species except for *P. forsythii*, ii) the (*P. erythrurus*, *P. vlangalii*) node, iii) the (*P. theobaldi*, *P. vlangalii* + *P. erythrurus*) node, and iv) the ancestral node for all *P. theobaldi*.

MtDNA sequences were partitioned as follows: the first and second codon positions of coding sequences (cp1&2), the third codon position of coding sequences (cp3) and tRNAs. The HKY + G DNA substitution model was used to account for within-partition heterogeneity because it is a relatively simple model suitable for all partitions (as assessed from log-likelihood scores for a specific topology).

We used the time of a geological event to calibrate divergence times on the tree. The last tectonic uplift of the middle and eastern Kunlun Mountains that separate the two species (*P. erythrurus*, *P. vlangalii*) (node ii) above) has been dated at 3.9 ± 0.6 Ma and 4.2 ± 0.8 Ma^40^. This has been supported by a more recent study that synthesized several different sources of data (low-temperature thermo-chronology data, sedimentary deposit, and structural deformation record) and suggested that last period of intensive uplift of these mountains started approximately 5 Ma^41^. We represented this vicariant event using a Uniform prior with upper and lower limits [3.3, 5.0], on the (*P. erythrurus*, *P. vlangalii*) node. It should be noted that Renner^13^ recently rejected the findings of several studies (including our own) that use QTP uplift events as calibrations in dating analyses, while accepting dates provided by studies that estimated times using specified substitution rates. Although this is ostensibly justified relative to the argument presented in that paper (i.e., that uplift is not the cause of recent diversification) we believe it is rather simplistic. It is better to assess the validity of a suitable calibration(s) through the substitution rate that it implies rather than the simple application of a global rate from another study. Under a global clock we found a pairwise uncorrected mean distance of 0.0736 between the relevant daughter lineages which corresponds to a substitution rate between 0.0074–0.0111 subs/site/Ma under our U [3.3, 5.0] calibration. This is similar to published substitution rates for similar ND1/tRNA/ND2 sequence for other vertebrates, including lizards see^42, 43^, justifying our use of the calibration. A wide prior Uniform [3.3, 20] was also placed on the root of the tree (this was in fact too wide to have much impact on the estimated divergence times of interest). Divergence was recent so a strict clock was most suitable for our dating analysis^44^.

Baseml^45^ was used to carry out a maximum likelihood dating analysis on the maximum clade credibility topology obtained from the posterior sample of trees in the BEAST analysis. The use of the HKY85 + G site model and a strict clock model provided similarity with the BEAST analysis. Time constraints were specified as single points that were equivalent to the means of the prior distributions described above. Unlike the Bayesian analysis, this analysis does not provide a reasonable estimate of the uncertainty associated with node age estimates^46, 47^. However, it has the advantage of providing divergence estimates that are not influenced by the prior on divergence times (which can strongly influence marginal posteriors when sequence divergence is low^47, 48^).

### Bayesian Phylogeographic and Ecological Clustering (BPEC) analyses

BPEC was used to determine coherent geographical clusters that were consistent with mtDNA relationships. The method can account for haplotype connection ambiguities due to the presence of loops and estimates posterior probabilities under a coalescent-based migration-mutation model^34^. BPEC represents an important advance over previous similar approaches that were often based on only one potentially ambiguously connected network.

We analysed all mtDNA haplotypes and their distributions based on latitudes and longitudes of the 60 sample sites (coordinates recorded in the field). After several preliminary trials, we carried out definitive analyses with the prior on the maximum number of migrations specified as 5 and without allowing relaxation of the parsimony criterion (in order to allow convergence). MCMC chains were run for 40 million steps, with 2000 posterior samples being saved for analysis.

### Species delimitation analyses

We applied the genetic species delimitation analysis that is implemented within the program BPP3.1^49, 50^, using phylogeographic clusters identified by BPEC as proposed species. This allowed examination of whether we could delimit the geographical groupings identified by BPEC. The analysis was based on the two nuclear markers. If the phylogeographic patterns represented by alleles from the two nuclear markers were statistically concordant with the mtDNA phylogeography, then this analysis should support species delimitation of individuals from different mtDNA clusters. Median-joining networks (software: Network 4.6.0.0, Fluxus Engineering) were used to visualize the relationships among the nuclear haplotypes. The use of only two loci could diminish the statistical power to delimit species. However we note that a posterior probability of 0.65 for a full delimitation was previously achieved using two loci in a five species analysis with just 17 individuals^50^, while our analysis used over 100 individuals (see later). In the first set of analyses, a guide tree was specified (from the mtDNA tree) to describe the hierarchical relationships among potential species identified by BPEC. Several analyses were conducted with different priors. Gamma distributions (G) with the following shape and scale parameters were used to specify the population size (θ_s_) and root divergence time (τ_0_) parameters: (i) θ_s_ G(1,1), τ_0_ G(1,1), (ii) θ_s_ G(1,10), τ_0_ G(1,10), (iii) θ_s_ G(1,10), τ_0_ G(1,1), (iv) θ_s_ G(1,1), τ_0_ G(1,10). The MCMC chain was run for 250,000 steps (following a burnin of 10000 steps), sampled every 50 steps. Each analysis was run three times to confirm consistency between runs.

A second set of 3 replicate analyses used no guide tree and therefore incorporated the uncertainty in the phylogenetic relationships among the clusters identified by BPEC. In these analyses the prior G(1, 1) was used for both θ_s_ and τ_0_ (this allowed considerable flexibility for both parameters).

## Electronic supplementary material

The geography and timing of genetic divergence in the lizard Phrynocephalus theobaldi on the Qinghai-Tibetan plateau
