# Supplementary material for: The geography and timing of genetic divergence in the lizard Phrynocephalus theobaldi on the Qinghai-Tibetan plateau
Source: Sci Rep. 2017 May 23;7:2281. doi: 10.1038/s41598-017-02674-4 (PMC5442162; doi:10.1038/s41598-017-02674-4)

Supplementary Information

Title

The geography and timing of genetic divergence in the lizard *Phrynocephalus theobaldi* on the Qinghai-Tibetan plateau

Author list

Yuanting Jin, Naifa Liu, Richard P. Brown

Supplementary Information S1

Sample sites, haplotypes and individuals of *Phrynocephalus theobaldi*, for mtDNA (a), *AME* (b) and *RAG-1* (c)

(a)

Sample site	Latitude (°)	Longitude (°)	MtDNA haplotype [specimen number]
1	29.4671	86.1068	40[11N0101]
2	29.4087	85.5703	35[11N0205,11N0204,11N0201,11N0202],36[11N0203],38[11N0207]
3	29.5690	85.8346	35[11N0305,11N0308,11N0302,11N0304,11N0310,11N0303,11N0301,11N0312,11N0309],39[11N0307,11N0311]
4	29.5004	84.5607	32[11N0402,11N0401,11N0406,11N0403,11N0405,11N0404,11N0408,11N0409,11N0407]
5	29.6653	84.1642	32[11N0508,11N0504,11N0505,11N0502,11N0506,11N0507,33[11N0503]
6	30.8682	83.7873	32[11N0602,11A0603]
7	30.7917	81.3781	71[11N0701,11N0702]
8	30.7247	81.3676	71[11N0803,11N0802,11N0801]
9	30.6855	81.3135	71[11N0902],72[11N0901]
10	30.3854	81.1539	73[11N1001]
11	31.4363	80.5239	67[11N1106,11N1105,11N1104,11N1102,11N1103],68[11N1101]
12	31.6625	80.3162	70[11N1204],69[11N1202,11N1203,11N1201]
13	32.1264	80.0801	69[11N1304,11N1308,11N1305,11N1306,11N1310,11N1307,11N1303,11N1302,11N1312,11N1309,11N1301], 67[11N1311]
14	32.3962	80.044	46[11N1401, 11N1402],50[11N1403]
15	32.3666	80.3671	45[11N1501],46[11N1502],59[11N1504,11N1506], 60[11N1503]
16	32.3660	80.7538	58[11N1602],79[11N1601]
17	32.4606	81.9143	61[11N1702]
18	32.5436	80.6392	80[11N1803]
19	32.5365	80.5477	46[11N1907,11N1906],58[11N1902, 11A1904,11N1905,11N1901,11N1909,11N1910,11N1908],63 [11N1903]

20	32.4138	79.7666	58[11N2007],64[11N2006,11N2003,11N2002,11N2004,11N2005]
21	32.4602	80.9279	46[11N2105,11N2104,11N2106,11N2103],53[11N2102],56[11N2107],57[11N2101]
22	33.7488	80.4482	80[11N2201,11N2205,11N2202],81[11N2204],82[11N2203]
23	33.6063	79.3203	76[11N2301],77[11N2302]
24	33.4593	79.8318	76[11N2403,11N2401,11N2402,11N2404]
25	33.5148	80.9059	76[11N2505,11N2502,11N2504,11N2506,11N2503]
26	33.1164	80.247	79[11N2606,11N2602,11N2605,11N2607,11N2604]
27	33.1874	80.8971	78[11N2703,11A2702],83[11N2701]
28	32.5832	80.0459	58[11N2802,11N2801]
29	32.5612	80.1197	46[11N2901,11N2904,11N2902,11N2905],58[11N2903],62[11N2906,11N2907]
30	32.4266	80.0114	46[11N3003,11N3006,11N3007,11N3009,11N3005,11N3014,11N3010,11N3017,11N3020,11N3011,11N3015]
			49[11N3002],54[11N3013],55[11N3016]
31	30.0300	83.4902	32[11N3103,11N3102,11N3101]
32	30.8853	83.7183	32[11N3203,11N3202,11N3201,RDQ6]
33	29.5650	85.8526	35[11N3304,11N3308,11N3307,11N3301],39[11N3306,11N3305,11N3303,11N3302],17[RDQ12],18[RDQ11]
34	29.9643	85.4416	37[11N3402],36[11N3401]
35	28.5932	87.0844	43[11N3505,11N3508,11N3511,11N3504,11N3501,11N3510,11N3503,11N3502,11N3507,11N3509, 11N3512],44[11N3514]
36	29.1659	87.6883	41[11N3601,11N3603],42[11N3602]
37	29.1516	88.62	6[11N3701,11N3703,11N3707,11N3705,11N3709],11[11N3706,11N3704],9[11N3708],14[11N3710]
38	29.3631	89.8471	7[11N3805,11N3802,11N3804,11N3803],8[11N3801]
39	29.3333	89.3377	5[11N3907],4[11N3906,11N3903],10[11N3905],12[11N3902,11N3904],13[11N3908,11N3901]
40	29.3795	90.8131	15[11N4001,11N4004,11N4003,11N4005],20[11N4006,11N4002]
41	29.3819	91.8627	15[11N4101]
42	29.2588	91.5336	19[N154],25[N155],28[N221,N220]
43	29.2573	91.596	29[N223],27[N222]
44	29.2825	91.8117	22[N157,N158,N156]

45	29.2388	92.0122	25[N159,N163]
46	29.1129	92.6462	25[N167,N166,N168,N164]
47	29.0839	92.7673	25[N176,N175],27[N172,N171,N173,N174,N178,N177]
48	29.9933	93.2489	25[N181]
49	29.3814	94.4155	25[N191]
50	29.3800	90.86	16[T275],15[T268,T269,T273,T277,T280N,T272,T267, T269B]
51	29.3200	91.82	22[T098, T100,T102,T103,T104,T106, T098B], 23[T099B T099], 24[T101]
52	29.2700	88.91	1[T151,T148,T153,T158,T160],2[T146,T154],3[T149,T145,T155,T156]
53	29.3200	85.23	32[T172,T171,T176,T177,T182,T186],34[T139,T163,T164,T166,T169,T170,T173,T175,T181,T183]
54	29.5700	84.85	35[T191,T192,T190]
55	29.8800	83.73	30[T196,T197,T198],32[T194,T199]
56	30.2800	82.95	31[T202],32[T200,T201,T203,T205,T206]
57	30.7400	81.64	71[T207,T208,T209,T210]
58	31.4900	80.44	65[T215,T233],66[T214,T221,T238],67[T212,T218,T213,T219,T222,T229,T230,T232,T236,T239,T241,T244, T240, T237,T224,T227,T211]
59	33.3900	79.74	74[T247],75[T252],76[T246,T249,T250,T245,T251,T253,T254,T255,T256,T259,T261,T262,T263,T264, T248]
60	32.5400	80.06	46[T291,T299,T300,T297], 47[T294,T295],48[T290,T298],51[T304], 52[T296,T301],58[T292,T293,T302,11N6004]

(b)

AME Haplotype	Individual
*AME*_Hap_1	T100,T101,T280N,T273,T272,T268,T267,11N4006,11N4004,11N4002,11N4001,11N3801,11N3802,11N3805,11N3901,11N3903,11N3904,11N3905,11N3908,11N3708,11N3703,11N3701,11N3603,11N1502,11N1504,11N1602,11N1904,11N2106,11N2102,11N2801,11N2904,11N2905,11N3009,11N3016,11N2607,11N2606,11N2703,11N2506,11N2204,11N2202,N220,N221
*AME*_Hap_2	11N3702,11N0901,11N0902
*AME*_Hap_3	11N3601
*AME*_Hap_4	11N3501,11N3504,11N3507,11N3509,11N3511,11N3512,11N3514,11N3303,11N0304,11N0305,11N0308,11N0309,11N0310,11N0311,11N0207,11N3401,11N0506,T196,11N3102,11N2503
*AME*_Hap_5	11N3301
*AME*_Hap_6	11N1102,11N1101,11N1106,11N1103,11N1104,11N1105,11N1203,11N1202,11N1201,T229,T224,T213,T211,11N1309,11N1303,11N1305,11N1310,11N1302,11N1304,11N1311,11N1301,11N1312,11N1901,11N2003,T238,11N2802,11N6004,11N3011,T292
*AME*_Hap_7	11N2604,11N2602,T256,T254,T259
*AME*_Hap_8	11N2401,11N2402

(c)

RAG-1 haplotype	Individual
*RAG-1*_Hap_1	T100,T101,T280N,T273,T268,T267,11N4006,11N4004,11N4002,11N4001,11N3801,11N3802,11N3805,11N3901,11N3903,11N3904,11N3908,11N3703,11N3701,11N3601,11N3603,11N3501,11N3504,11N3507,11N3509,11N3511,11N3512,11N3514,11N0308,11N0309
*RAG-1*_Hap_2	T272
*RAG-1*_Hap_3	11N3905
*RAG-1*_Hap_4	11N3708
*RAG-1*_Hap_5	11N3702,11N0901,11N0902
*RAG-1*_Hap_6	11N3301,11N3303,11N0304,11N0310,11N0311,11N0207,11N3401
*RAG-1*_Hap_7	11N0305
*RAG-1*_Hap_8	11N0506,T196
*RAG-1*_Hap_9	11N3102
*RAG-1*_Hap_10	11N1102,11N1101,11N1106,11N1103,11N1104,11N1105,11N1202,11N1201,T224,T213,T211,11N1312,11N1904,11N2607,11N2604,11N2602,11N2703,11N2401,11N2402,11N2204,11N2202,T256,T254,T259
*RAG-1*_Hap_11	11N1203,11N1309,11N1303,11N1305,11N1310,11N1302,11N1304,11N1311,11N1301,11N1502,11N1504,11N1901,11N2003,11N2802,11N2904,11N3009,11N6004,T292
*RAG-1*_Hap_12	T229,T238
RAG-1_Hap_13	11N1602,11N2106,11N2102,11N2801,11N2905,11N3011,11N3016
*RAG-1*_Hap_14	11N2606,11N2503,11N2506
*RAG-1*_Hap_15	N220,N221

Supplementary Information S2
DNA sequences for the *P. theobaldi* haplotypes

The 83 mtDNA haplotypes (aligned) detected in *P. theobaldi*
1
GGCTGAACCATTATAATCATAGCCCTCTCCCCAAATCTAGCAATTATAAATATCTTTA
TCTACATTATTATAACCACCCCAATTTTTTGACAATAACAAACATATCAACAAAAA
CACTACAAAATTTAACCACAACATGAACAACCTCTATAACAATAACCCTTTCCACT
ACCTTCCTAATACTATCAACCAGCGGCCTACCACCGTTTACAGGATTTATACCAAAA
ATACTAATTCTTAATGAACTTATTTCACAAAAACTTACAGCACTAGCAACCCTAGCA
ATTATAACATCACTAATTAGTTTATTATTTTACTTACGAATCACATACCTAATTATAAT
ACTCACATCACCAACAACAACCACATCATCAACAAAATGACGAGCCCAAAACCAA
AAATTACAACTAATAACAATAATAATTCCAACAGCACTATTCATTACTCACCTAACC
CCAGCAATCCCGTTCTAAAGAAGCTTAGGATT-AAACTTATTAAACCGGTGGCCTTC
AACGCCACAAACAAGGGGG-AGCCCTTAGCTTCTGAAAGACCTATAGAACTTTCTT
CTACATCAT
2
GGCTGAACCATTATAATCATAGCCCTCTCCCCAAATCTAGCAATTATAAATATCTTTA
TCTACATTATTATAACCACCCCAATTTTTTAACAATAACAAACATATCAACAAAAA
CACTACAAAATTTAACCACAACATGAACAACCTCTATAACAATAACCCTTTCCACT
ACCTTCCTAATACTATCAACCAGCGGCCTACCACCGTTTACAGGATTTATACCAAAA
ATACTAATTCTTAATGAACTTATTTCACAAAAACTTACAGCACTAGCAACCCTAGCA
ATTATAACATCACTAATTAGTTTATTATTTTACTTACGAATCACATACCTAATTATAAT
ACTCACATCACCAACAACAACCACATCATCAACAAAATGACGAGCCCAAAACCAA
AAATTACAACTAATAACAATAATAATTCCAACAGCACTATTCATTACTCACCTAACC
CCAGCAATCCCGTTCTAAAGAAGCTTAGGATT-AAACTTATTAAACCGGTGGCCTTC
AACGCCACAAACAAGGGGGGAGCCCTTAGCTTCTGAAAGACCTATAGAACTTTCT
TCTACATCAT
3
GGCTGAACCATTATAATCATAGCCCTCTCCCCAAATCTAGCAATTATAAATATCTTTA
TCTACATTATTATAACCACCCCAATTTTTTAACAATAACAAGCATATCAACAAAAA
CACTACAAAATTTAACCACAACATGAACAACCTCTATAACAATAACCCTTTCCACT
ACCTTCCTAATACTATCAACCAGCGGCCTACCACCGTTTACAGGATTTATACCAAAA
ATACTAATTCTTAATGAACTTATTTCACAAAAACTTACAGCACTAGCAACCCTAGCA
ATTATAACATCACTAATTAGTTTATTATTTTACTTACGAATCACATACCTAATTATAAT
ACTCACATCACCAACAACAACCACATCATCAACAAAATGACGAGCCCAAAACCAA
AAATTACAACTAATAACAATAATGATTCCAACAGCACTATTCATTACTCACCTAACC
CCAGCAATCCCGTTCTAAAGAAGCTTAGGATT-AAACTTATTAAACCGGTGGCCTTC
AACGCCACAAACAAGGGGGGAGCCCTTAGCTTCTGAAAGACCTATAGAACTTTCT
TCTACATCAT
4
GGCTGAACCATTATAATCATAGCCCTCTCCCCAAATCTAGCAATTATAAATATCTTCA
TCTACATTATTATAACCACCCCAATTTTTTAACAATAACAAACATATCAACAAAAA
CACTACAAAATTTAACCACAACATGAACAACCTCTATAACAATAACCCTTTCCACT

ACCTTCCTAATACTATCAACCAGCGGCCTACCACCGTTTACAGGGTTTATACCAAAA
ATACTAATTCTTAATGAACTTATTTCACAAAAACTTACAGCACTAGCAACCCTAGCA
ATTATAACATCACTAATTAGTTTATTATTTTACTTACGAATCACATACCTAATTATAAT
ACTTACATCACCAACAACAACCACATCATCAACAAAATGACGAGCCCAAAACCAA
AAATTACAACTAATAACAATAATAATTCCAACAGCACTATTCATTACTCACCTAACC
CCAGCAATCCCGTTCTAAAGAAGCTTAGGATT-AAACTTATTAAACCGGTGGCCTTC
AACGCCACAAACAAGGGGG-AGCCCTTAGCTTCTGAAAGACCTATAGAACTTTCTT
CTACATCAT

5

GGCTGAACCATTATAATCATAGCCCTCTCCCCAAATCTAGCAATTATAAATATCTTCA
TCTACATTATTATAACCACCCCAATTTTTTTAACAATAACAAACATATCAACAAAAA
CACTACAAAATTTAACCACAACATGAACAACCTCTATAACAATAACCCTTTCCACT
ACCTTCCTAATACTATCAACCAGCGGCCTACCGCCGTTTACAGGGTTTATACCAAAA
ATACTAATTCTTAATGAACTTATTTCACAAAAACTTACAGCACTAGCAACCCTAGCA
ATTATAACATCACTAATTAGTTTATTATTTTACTTACGAATCACATACCTAATTATAAT
ACTTACATCACCAACAACAACCACATCATCAACAAAATGACGAGCCCAAAACCAA
AAATTACAACTAATAACAATAATAATTCCAACAGCACTATTCATTACTCACCTAACC
CCAGCAATCCCGTTCTAAAGAAGCTTAGGATT-AAACTTATTAAACCGGTGGCCTTC
AACGCCACAAACAAGGGGG-AGCCCTTAGCTTCTGAAAGACCTATAGAACTTTCTT
CTACATCAT

6

GGCTGAACCATTATAATCATAGCCCTCTCCCCAAATCTAGCAATTATAAATATCTTCA
TCTACATTATTATAACCACCCCAATTTTTTTAACAATAACAAACATATCAACAAAAA
CACTACAAAATTTAACCACAACATGAACAACCTCTATAACAATAACCCTTTCCACT
ACCTTCCTAATACTATCAACCAGCGGCCTACCACCGTTTACAGGATTTATACCAAAA
ATACTAATTCTTAATGAACTTATTTCACAAAAACTTACAGCACTAGCAACCCTAGCA
ATTATAACATCACTAATTAGTTTATTATTTTACTTACGAATCACATACCTAATTATAAT
ACTTACATCACCAACAACAACCACATCATCAACAAAATGACGAGCCCAAAACCAA
AAATTACAACTAATAACAATAATAATTCCAACAGCACTATTCATTACTCACCTAACC
CCAGCAATCCCGTTCTAAAGAAGCTTAGGATT-AAACTTATTAAACCGGTGGCCTTC
AACGCCACAAACAAGGGGGGAGCCCTTAGCTTCTGAAAGACCTATAGAACTTTCT
TCTACATCAT

7

GGCTGAACCATTATAATCATAGCCCTCTCCCCAAATCTAGCAATTATAAATATCTTCA
TCTACATTATTATAACCACCCCAATTTTTTTAACAATAACAAACATATCAACAAAAA
CACTACAAAATTTAACCACAACATGAACAACCTCTATAACAATAACCCTTTCCACT
ACCTTCCTAATACTATCAACCAGCGGCCTACCACCGTTTACAGGATTTATACCAAAA
ATACTAATTCTTAATGAACTTATTTCACAAAAACTTACAGCACTAGCAACCCTAGCA
ATTATAACATCACTAATTAGTTTATTATTTTACTTACGAATCACATACCTAATTATAAT
ACTTACATCACCAACAACAACCACATCATCAACAAAATGACGAGCCCAAAACCAA
AAATTACAACTAATAACAATAATAATTCCAACAGCACTATTCATTACTCACCTAACC
CCAGCAATCCCGTTCTAAAGAAGCTTAGGATT-AAGCTTATTAAACCGGTGGCCTTC
AACGCCACAAACAAGGGGGGAGCCCTTAGCTTCTGAAAGACCTATAGAACTTTCT
TCTACATCAT

8
GGCTGAACCATTATAATCATAGCCCTCTCCCCAAATCTAGCAATTATAAATATCTTCA
TCTACATTATTATAACCACCCCAATTTTTTAACAATAACAAACATATCAACAAAAA
CACTACAAAATTTAACCACAACATGAACAACCTCTATAACAATAACCCTTTCCACT
ACCTTCCTAATACTATCAACCAGCGGTCTACCACCGTTTACAGGATTTATACCAAAA
ATACTAATTCTTAATGAACTTATTTCACAAAAACTTACAGCACTAGCAACCCTAGCA
ATTATAACATCACTAATTAGTTTATTATTTTACTTACGAATCACATACCTAATTATAAT
ACTTACATCACCAACAACAACCACATCATCAACAAAATGACGAGCCCAAAACCAA
AAATTACAACTAATAACAATAATAATTCCAACAGCACTATTCATTACTCACCTAACC
CCAGCAATCCCGTTCTAAAGAAGCTTAGGATT-AAGCTTATTAAACCGGTGGCCTTC
AACGCCACAAACAAGGGGGGAGCCCTTAGCTTCTGAAAGACCTATAGAACTTTCT
TCTACATCAT
9
GGCTGAACCATTATAATCATAGCCCTCTCCCCAAATCTAGCAATTATAAATATCTTCA
TCTACATTATTATAACCACCCCAATTTTTTAACAATAACAAACATATCAACAAAAA
CACTACAAAATTTAACCACAACATGAACAACCTCTATAACAATAACCCTTTCCACT
ACCTTCCTAATACTATCAACCAGCGGCCTACCACCGTTTACAGGATTTATACCAAAA
ATACTAATTCTTAGTGAACTTATTTCACAAAAACTTACAGCACTAGCAACCCTAGCA
ATTATAACATCACTAATTAGTTTATTATTTTACTTACGAATCACATACCTAATTATAAT
ACTTACATCACCAACAACAACCACATCATCAACAAAATGACGAACCCAAAACCAA
AAATTACGACTAATAACAATAATAATTCCAACAGCACTATTCATTACTCACCTAACC
CCAGCAATCCCATTCTAAAGAAGCTTAGGATT-AAACTTATTAAACCGGTGGCCTTC
AACGCCACAAACAAGGGGGGAGCCCTTAGCTTCTGAAAGACCTATAGAACTTTCT
TCTACATCAT
10
GGCTGAACCATTATAATCATAGCCCTCTCCCCAAATCTAGCAATTATAAATATCTTCA
TCTACATTATTATAACCACCCCAATTTTTTAACAATAACAAACATATCAACAAAAA
CACTACAAAATTTAACCACAACATGAACAACCTCTATAACAATAACCCTTTCCACT
ACCTTCCTAATACTATCAACCAGCGGCCTACCACCGTTTACAGGATTTATACCAAAA
ATACTAATTCTTAATGAACTTATTTCACAAAAACTTACAGCACTAGCAACCCTAGCA
ATTATAACATCACTAATTAGTTTATTATTTTACTTACGAATCACATACCTAATTATAAT
ACTTACATCACCAACAACAACCACATCGTCAACAAAATGACGAACCCAAAACCAA
AAATTACAACTAATAACAATAATAATTCCAACAGCACTATTCATTACTCACCTAACC
CCAGCAATCCCGTTCTAAAGAAGCTTAGGATT-AAACTTATTAAACCGGTGGCCTTC
AACGCCACAAACAAGGGGG-AGCCCTTAGCTTCTGAAAGACCTATAGAACTTTCTT
CTACATCAT
11
GGCTGAACCATTATAATCATAGCCCTCTCCCCAAATCTAGCAATTATAAATATCTTCA
TCTACATTATTATAACCACCCCAATTTTTTAACAATAACAAACATATCAACAAAAA
CACTACAAAATTTAACCACAACATGAACAACCTCTATAACAATAACCCTTTCCACT
ACCTTCCTAATACTATCAACCAGCGGCCTACCACCGTTTACAGGATTTATACCAAAA
ATACTAATTCTTAATGAACTTATTTCACAAAAACTTACAGCACTAGCAACCCTAGCA
ATTATAACATCACTAATTAGTTTATTATTTTACTTACGAATCACATACCTAATTATAAT
ACTTACATCACCAACAACAACCACATCATCAACAAAATGACGAGCCCAAAACCAA

AAATTACAACTAATAACAATAATAATTCCAACAGCACTATTCATTACTCACCTAACC
CCAGCAATCCCGTTCTAAAGAAGCTTAGGATT-AAACTTATTAAACCGGTGGCCTTC
AACGCCACAAACAAGGGGG-AACCCTTAGCTTCTGAAAGACCTATAGAACTTTCTT
CTACATCAT
12
GGCTGAACCATTACAATCATAGCCCTCTCCCCAAATCTAGCAATTATAAATATCTTCA
TCTACATTATTATAACCACCCCAATTTTTTAACAATAACAAATATATCAACAAAAAC
ACTACAAAATTTAACCACAACATGAACAACCTCTATAACAATAACCCTTTCCACTA
CCTTCCTAATACTATCAACCAGCGGCCTACCGCCATTTACAGGATTTATACCAAAAA
TACTAATTCTTAATGAACTTATTTCACAAAAACTTACAGCACTAGCAACCCTAGCAA
TTATAACATCACTAATTAGTTTATTATTTTACTTACGAATCACATACCTAATTATAATA
CTTACATCACCAACAACAACCACATCATCAACAAAATGACGAGCCCAAAACCAAA
AATTACAACTAATAACAATAATAATTCCAACAGCACTATTCATTACTCACCTAACCC
CAGCAATCCCGTTCTAAAGAAGCTTAGGATA-AAACTTAATAAACCGGTGGCCTTC
AACGCCACAAACAAGGTGGGAGCCCTTAGCTTCTGAAAGACCTATAGAACTTTCT
CCTACATCAT
13
GGCTGAACCATTACAATCATAGCCCTCTCCCCAAATCTAGCAATTATAAATATCTTCA
TCTACATTATTATAACCACCCCAATTTTTTAACAATAACAAATATATCAACAAAAAC
ACTACAAAATTTAACCACAACATGAACAACCTCTATAACAATAACCCTTTCCACTA
CCTTCCTAATACTATCAACCAGCGGCCTACCGCCATTTACAGGATTTATACCAAAAA
TACTAATTCTTAATGAACTTATTTCACAAAAACTTACAGCACTAGCAACCCTAGCAA
TTATAACATCACTAATTAGTTTATTATTTTACTTACGAATCACATACCTAATTATAATA
CTTACATCACCAACAACAACCACATCATCAACAAAATGACGAGCCCAAAACCAAA
AATTACAACTAACAACAATAATAATTCCAACAGCACTATTCATTACTCACCTAACCC
CAGCAATCCCGTTCTAAAGAAGCTTAGGATA-AAACTTATTAAACCGGTGGTTTTCA
ACGCCACAAACAAGGTGGGAGCCCTTAGCTTCTGAAAGACCTATAGAACTTTCTC
CTACATCAT
14
GGCTGAACCATTATAATCATAGCCCTCTCCCCAAATCTAGCAATTATAAATATCTTCA
TCTACATTATTATAACCACCCCAATTTTTTAACAATAACAAACATATCAACAAAAA
CACTACAAAATTTAACCACAACATGAACAACCTCTATAACAATAACCCTTTCCACT
ACCTTCCTAATACTATCAACCAGCGGCCTACCACCATTTACAGGATTTATACCAAAA
ATACTAATTCTTAATGAACTTATTTCACAAAAACTTACAGCACTAGCAACCCTAGCA
ATTATAACATCACTAATTAGTTTATTATTTTACTTACGAATCACGTACCTAATTATAAT
ACTTACATCACCAACAACAACCACATCATCAACAAAATGACGAGCCCAAAACCAA
AAATTACAACTAATAACGATAATAATTCCAACAGCACTATTCATTACTCACCTAACC
CCAGCAATCCCGTTCTAAAGAAGCTTAGGATT-AAACTTATTAAACCGGTGGCCTTC
AACGCCACAAACAAGGTGGGAGCCCTTAGCTTCTGAAAGACCTATAGAACTTTCT
CCTACATCAT
15
GGCTGAACCACCATAATCATAGCCCTCTCCCCAAATCTAGCAATTATAAATATTTTC
ACTTACATTATTATAACTACCCCAATTTTTTAATAATAACAAATATATCAACAAAAA
CACTACAAAACTTAACCACAACATGAACAACCTCTGCAACAATAACCCTCTCCACT

GCCTTCCTAATATTATCAACCAGCGGCCTACCGCCATTCACAGGATTTATACCAAAA
ATACTAATTCTTAATGAACTTATTTCACAAAAACTCACAACACTAGCAGCTCTAGCA
ATTATAACATCACTAATTAGTTTATTATTTTACTTACGAATTACATACCTAATTATAATA
CTTACATTACCAACAACAACCACAGCATCAACAAAATGACGAGCCCAAAACCAAA
AATTACAACTAACAACAATAATAATTCCAACAGCACTATTCACTACTCACCTAATCC
CAGCAATCCCATTCTAAAGAAGCTTAGGATT-AAACTCATTAAACCAGTGGCCTTCA
ACACCACAAATAAGGGAA-AACCCTTAGCTTCTGAAAGACCTATAGAACTTTCTCC
TACATCAT

16

GGCTGAACCACCATAATCATAGCCCTCTCCCCAAATCTAGCAATTATAAATATTTTC
ACTTACATTATTATAACTACCCCAATTTTTTAATAATAACAAATATATCAACAAAAA
CACTACAAAACTTAACCACAACATGAACAACCTCTGCAACAATAACCCTCTCCACT
GCCTTCCTAATATTATCAACCAGCGGCCTACCGCCATTCACAGGATTTATACCAAAA
ATACTAATTCTTAATGAACTTATTTCACAAAAACTCACAACACTAGCAACTCTAGCA
ATTATAACATCACTAATTAGTTTATTATTTTACTTACGAATTACATACCTAATTATAATA
CTTACATTACCAACAACAACCACAGCATCAACAAAATGACGAGCCCAAAACCAAA
AATTACAACTAACAACAATAATAATTCCAACAGCACTATTCACTACTCACCTAATCC
CAGCAATCCCATTCTAAAGAAGCTTAGGATT-AAACTCATTAAACCAGTGGCCTTCA
ACACCACAAATAAGGGAA-AACCCTTAGCTTCTGAAAGACCTATAGAACTTTCTCC
TACATCAT

17

GGCTGAACCACCATAATCATAGCCCTCTCCCCAAATCTAGCAATTATAAATATTTTC
ACTTACATTATTATAACTACCCCAATTTTTTAATAATAACAAATATATCAACAAAAA
CACTACAAAACTTAACCACAACATGAACAACCTCTGCAACAATAACCCTCTCCACT
GCCTTCCTAATATTATCAACCAGCGGCCTACCGCCATTCACAGGATTTATACCAAAA
ATACTAATTCTTAATGAACTTATTTCACAAAAACTCACAACACTAGCAGCTCTAGCA
ATTATAACATCACTAATTAGTTTATTATTTTACTTACGAATTACATACCTAATTATAATA
CTTACATTACCAACAACAACCACAGCATCAACAAAATGACGAGCCCAAAACCAAA
AATTACAACTAACAACAATAATAATTCCAACAGCACTATTCACTACTCACCTAATCC
CAGCAATCCCATTCTAAAGAAGCTTAGGATT-AAACTCATTAAACCAGTGGCCTTCA
ACACCACAAACAAGGGAA-AACCCTTAGCTTCTGAAAGACCTATAGAACTTTCTCC
TACATCAT

18

GGCTGAACCACCATAATCATAGCCCTCTCCCCAAATCTAGCAATTATAAATATTTTC
ACTTACATTATTATAACTACCCCAATTTTTTAATAATAACAAATATATCAACAAAAA
CACTACAAAACTTAACCACAACATGAACAACCTCTGCAACAATAACCCTCTCCACT
GCCTTCCTAATATTATCAACCAGCGGCCTACCACCATTCACAGGATTTATACCAAAA
ATACTAATTCTTAATGAACTTATTTCACAAAAACTCACAACACTAGCAGCTCTAGCA
ATTATAACATCACTAATTAGTTTATTATTTTACTTACGAATTACATACCTAATTATAATA
CTTACATTACCAACAACAACCACAGCATCAACAAAATGACGAACCCAAAACCAAA
AATTACAACTAACAACAATAATAATTCCAACAGCACTATTCACTACTCACCTAATCC
CAGCAATCCCATTCTAAAGAAGCTTAGGATT-AAACTCATTAAACCAGTGGCCTTCA
ACACCACAAACAAGGGAA-AACCCTTAGCTTCTGAAAGACCTATAGGACTTTCTCC
TACATCAT

19
GGCTGAACCACCATAATCATAGCCCTCTCCCCAAATCTAGCAATTATAAATATTTTC
ACTTACATTATTATAACTACCCCAATTTTTTAATAATAACAAATATATCAACAAAAA
CACTACAAAACTTAACCACAACATGAACAACCTCTGCAACAATAACCCTCTCCACT
GCCTTCCTAATATTATCAACCAGCGGCCTACCACCATTCACAGGATTTATACCAAAA
ATACTAATTCTTAATGAACTTATTTCACAAAAACTCACAACACTAGCAGCTCTAGCA
ATTATAACATCACTAATTAGTTTATTATTTTACTTACGAATTACATACCTAATTATAATA
CTTACATTACCAACAACAACCACAGCATCAACAAAATGACGAACCCAAAACCAAA
AATTACAACTAACAACAATAATAATTCCAACAGCACTATTCACTACTCACCTAATCC
CAGCAATCCCGTTCTAAAGAAGCTTAGGATT-AAACTCATTAAACCAGTGGCCTTC
AACACCACAAACAAGGGAA-AACCCTTAGCTTCTGAAAGACCTATAGGACTTTCTC
CTACATCAT
20
GGCTGAACTATCATAATCATAGCCCTCTCCCCAAATCTAGCAATTATAAATATTTTCA
CTTACATTATTATAACTACCCCAATTTTTTAATAATAACAAATATATCAACAAAAAC
ACTACAAAACTTAACCACAACATGAACAACCTCTGCAACAATAACCCTCTCCACT
GCCTTCCTAATATTATCAACCAGCGGCCTACCGCCATTCACAGGATTTATACCAAAA
ATACTAATTCTTAATGAACTTATTTCACAAAAACTCACAACACTAGCAGCTCTAGCA
ATTATAACATCACTAATTAGTTTATTATTTTACTTACGAATTACATACCTAATTATAATA
CTTACATCACCAACAACAACCACAGCATCAACAAAATGACGAACCCAAAACCAAA
AATTACAACTAACAACAATAATAATTCCAACAGCACTATTCACTACTCACCTAATCC
CAGCAATCCCGTTCTAAAGAAGCTTAGGATT-AAACTCATTAAACCAGTGGCCTTC
AACACCACAAACAAGGGAA-AACCCTTAGCTTCTGAAAGACCTATAGGACTTTCTC
CTACATCAT
21
GGCTGAACCACCATAATCATAGCCCTCTCCCCAAATCTAGCAATTATAAATATTTTTA
CTTACATTATTATAACCACCCCAATTTTTTAATAATAACAAATATATCAACAAAAAC
ACTACAAAATTTAACCACAACATGAACAACCTCTGCAACAATAACCCTCTCCACTG
CCTTCCTAATGTTATCAACCAGCGGCCTACCACCATTCACAGGATTTATACCAAAAA
TACTAATTCTTAATGAACTTATTTCACAAAAACTCACAACACTAGCAGCTCTAGCAA
TTATAACATCACTAATTAGTTTATTATTTTACTTACGAATCACATACCTAATTATAATA
CTTACATCACCAACAACAACCACAGCATCAACAAAATGACGAGCCCAAAACCAAA
AATTACAACTAACAACAATAATAATTCCAACAGCACTGTTCACTACTCACCTAATCC
CAGCAATCCCATTCTAAAGAAGCTTAGGATT-AAACTTATTAAACCAGTGGCCTTCA
ACACCACAAACAAGGGAA-AACCCTTAGCTTCTGAAAGACCTATAGGACTTTCTCC
TACATCAT
22
GGCTGAACCATCATAATCATAGCCCTATCCCCAAATCTAGCAATTATAAATATTTTTA
CTTACATTATTATAACCACCCCAATTTTTTAATAATAACAAATATATCAACAAAAAC
GCTACAAAACTTAACCACAACATGAACAACCTCTGCAACAATAACCCTATCCACTA
CCTTCCTAATACTATCAACCAGCGGCCTACCACCATTCACAGGATTTATACCAAAAA
TACTAATTCTTAATGAACTTATTTCACAAAAACTCACAATACTAGCAGCTCTAGCAA
TTATAACATCACTAATTAGTTTATTATTTTACTTACGAATTACATACCTAATTATAATAC
TTACATCACCAACAACAACCACAGCATCAACAAAATGACGAGCCCAAAACCAAA

AATTACAACTAACAACAATAATAATTCCAACAGCACTATTCACTATTCACCTAATCC
CAGCAATCCCGTTCTAAAGAAGCTTAGGATT-AAGCTTATTAAACCAGTGGCCTTCA
ACACCACAAACAAGGGAA-AACCCTTAGCTTCTGAAAGACCTATAGGACTTTCTCC
TACATCAT
23
GGCTGAACCATCATAATCATAGCCCTATCCCCAAATCTAGCAATTATAAATATTTTTA
CTTACATTATTATAACCACCCCAATTTTTTAATAATAACAAATATATCAACAAAAAC
GCTACAAAACTTAACCACAACATGAACAACCTCTGCAACAATAACCCTATCCACTA
CCTTCCTAATACTATCAACCAGCGGCCTACCACCATTCACAGGATTTATACCAAAAA
TACTAATTCTTAATGAACTTATTTCACAAAAACTCACAATACTAGCAGCTCTAGCAA
TTATAACATCACTAATTAGTTTATTATTTTACTTACGAATTACATACCTAATTATAATAC
TTACATCACCAACAACAACCACAGCATCAACAAAATGACGAGCCCAAAACCAAA
AATTACAACTAACAACAATAATAATTCCAACAGCACTATTCACTATTCACCTAATCC
CAGCAATCCCATTCTAAAGAAGCTTAGGATT-AAGCTTATTAAACCAGTGGCCTTCA
ACACCACAAACAAGGGAA-AACCCTTAGCTTCTGAAAGACCTATAGGACTTTCTCC
TACATCAT
24
GGCTGAACCACCATAATCATAGCCCTATCCCCAAATCTAGCAATTATAAATATTTCCA
CCTACATTATTATAACCACCCCAATTTTTTAATAATAACAAATATATCAACAAAAAC
GCTACAAAACTTAACCACAACATGAACAACCTCTGCAACAATAACCCTATCCACTA
CCTTCCTAATACTATCAACCAGCGGCCTACCACCATTCACAGGATTTATACCAAAAA
TACTAATTCTTAATGAACTTATTTCACAAAAACTCACAATACTAGCAGCTCTAGCAA
TTATAACATCACTAATTAGTTTATTATTTTACTTACGAATTACATACCTAATTATAATAC
TTACATCACCAACAACAACCACAGCATCAACAAAATGACGAGCCCAAAACCAAA
AATTACAACTAACAACAATAATAATTCCAACAGCACTATTCACTATTCACCTAATCC
CAGCAATCCCGTTCTAAAGAAGCTTAGGATT-AAGCTTATTAAACCAGTGGCCTTCA
ACACCACAAATAAGGGAA-AACCCTTAGCTTCTGAAAGACCTATAGGACTTTCTCC
TACATCAT
25 ??????????????????ATAGCCCTCTCCCCAAATCTAGCAATTATAAATATTTTC
ACTTACATTATTATAACCACCCCAATTTTTTAATAATAACAAATATATCAACAAAAA
CACTACAAAACTTAACCACAACTTGAACAACCTCTGCAACAATAACCCTATCTACT
ACCTTCCTAATACTATCAACCAGCGGCCTACCACCTTTCACAGGATTTATACCAAAA
ATACTAATTCTTAATGAACTTATTTCACAAAAACTCACAACACTAGCAGCTCTAGCA
ATTATAACATCACTAATTAGTTTATTATTTTACTTACGAATTACATACCTAATTATAATA
CTTACATCACCAACAACAACCACAGCATCAACAAAATGACGAGCCCAAAACCAAA
AATTACAACTAACAACAATAATAATTCCAACAGCACTATTCACTACTCACCTAATCC
CAGCAATCCCGTTCTAAAGAAGCTTAGGACT-AAGCTTATTAAACCAGTGGCCTTC
AACACCACAAACAAGGGAA-AACCCTTAGCTTCTGAAAGACCTATAGGACTTTCTC
CTACATCAT
26 ??????????????????ATAGCCCTCTCCCCAAATCTAGCAATTATAAATATTTTC
ACTTACATTATTATAACCACCCCAATTTTTTAATAATAACAAATATATCAACAAAAA
CACTACAAAACTTAACCACAACTTGAACAACCTCTGCAACAATAACCCTATCTACT
ACCTTCCTAATACTATCAACCAGCGGCCTACCACCTTTCACAGGATTTATACCAAAA
ATACTAATTCTTAATGAACTTATTTCACAAAAACTCACAACACTAGCAGCTCTAGCA

ATTATAACATCACTAATTAGTTTATTATTTTACTTACGAATTACATACCTAATTATAATA
CTTACATCACCAACAATAACCACAGCATCGACAAAATGACGAGCCCAAAACCAAA
AATTACAACTAACAACAATAATAATTCCAACAGCACTATTCACTACTCACCTAATCC
CAGCAATCCCGTTCTAAAGAAGCTTAGGACT-AAGCTTATTAAACCAGTGGCCTTC
AACACCACAAACAAGGGAA-AACCCTTAGCTTCTGAAAGACCTATAGGACTTTCTC
CTACATCAT

27 ??????????????????ATAGCCCTCTCCCCAAATCTAGCAATTATAAATATTTTC
ACTTACATTATTATAACCACCCCAATTTTTTTAATAATAACAAATATATCAACAAAAA
CACTACAAGACTTAACCATAACTTGAACAACCTCTGCAACAATAACCCTATCCACT
ACCTTCCTAATACTATCAACCAGTGGCCTACCACCCTTCACAGGATTTATACCAAAA
ATACTAATTCTTAATGAACTTATTTCACAAAAACTCACAACACTAGCAGCTCTAGCA
ATTATAACATCACTAATTAGTTTATTATTTTACTTACGAATTACATACCTAACTATAAT
ACTTACATCACCAACAACAACCACAGCATCAACAAAATGACGAACCCAAAACCAA
AAACTACAACTAACAACAATAATAATTCCAACAGCACTATTCACTACTCACCTAATC
CCAGCAATCCCGTTCTAAAGAAGCTTAGGACT-AAGCTTATTAAACCAGTGGCCTTC
AACACCACAAACAAGGGAA-AACCCTTAGCTTCTGAAAGACCTATAGGGCTTTCTC
CTACATCAT

28 ??????????????????ATAGCCCTCTCCCCAAATCTAGCAATTATAAATATCTGC
ACTTACATTATTATAACTACCCCAATTTTTTTAATAATAACAAACATATCAACAAAAA
CACTACAAAATTTAACCACAACATGAACAACCTCTACAGCAACAACCCTATCCATT
GCCCTCCTAATACTATCAACCAGCGGCCTACCACCATTCACAGGGTTTATACCAAA
AATACTAATTCTTAATGAGCTTATTTCACAAAAACTTACAACGCTAGCAACCCTAGC
AATTATAACATCACTAATTAGCTTAATATTTTACTTACGAATCACATACCTAATTATAA
TACTCACATCACCAATAACAACCCCATCATCAACAAAATGACGAACCCAAATCCAA
CAAACACAACCAATAACAATAATAATTCCAACAGCACTATTTATTACCCACCTAATC
CCAGCAATCCCGTTCTAAAGAAGCTTAGGATT-AAGTTTATTAAACCAGTGGCCTTC
AACACCACAAACAAGGGAA-AACCCTTAGCTTCTGAAAGACCTATAGGACTTTCTC
CTACATCAT

29 ??????????????????ATAGCCCTTTCCCCAAATCTAGCAATTATAAATATCTGC
ACTTACATTATTATAACTACCCCAATTTTTTTAATAATAACAAACATATCAACAAAAA
CACTACAAAATTTAACCACAACATGAACAACCTCTGCAGCAACAACCCTATCCATT
GCCCTCCTAATACTATCAACCAGCGGCCTACCACCATTCACAGGGTTTATACCAAA
AATACTAATTCTTAATGAGCTTATTTCACAAAAACTTACAACGCTAGCAACCCTAGC
AATTATAACATCACTAATTAGCTTATTATTTTACTTACGAATCACATACCTAATTATAA
TACTCACATCACCAATAACAACCCCATCATCAACAAAATGACGAACCCAAATCCAA
CAAACACAACCAATAACAATAATAATTCCAACAGCACTATTTATTACCCACCTAATC
CCAGCAATCCCGTTCTAAAGAAGCTTAGGATT-AAGTTTATTAAACCAGTGGCCTTC
AACACCACAAACAAGGGAA-AACCCTTAGCTTCTGAAAGACCTATAGGACTTTCTC
CTACATCAT

30
GGCTGAACCATTATAACCATAGCCATCTCCCCAAACGTAGCAATCATAAATATCTCC
ATTTACATTATTATGACTACTCCAATTTTTTTAATAATAACAAACACATCAACAAAAA
CACTACAAAATTTAACCACAACATGAACAACCTCTACAGCAACAACCCTCTCCATT
GCCCTCCTAATACTATCAACCAGCGGCCTACCACCATTCACAGGATTCATACCAAA

AATATTAATTCTTAATGAACTCATTTCACAAAAACTTACAACACTAGCAACCCTAGC
AATTATAACATCACTAGTTAGCTTATTATTTTACTTACGAATTACATACCTAATTATAA
TACTCACACCACCAATAACAACCCAATCATCAACAAAATGACGAACCCAAAACCA
AAAATCACAACTAATAACAATAATAACTCCAACAGCACTATTTATCACCCACCTAAT
CCCAGCAATCCCGTTCTAAAGAAGCTTAGGATT-AAGCTTATTAAACCAGTGGCCTT
CAACACCACAAACAAGGGAA-AACCCTTAGCTTCTGAAAGACCTATAGGACTTTCT
CCTACATCAT

31

GGCTGAACCATTATAACCATAGCCATCTCCCCAAACGTAGCAATCATAAATATCTCC
ATTTACATTATTATAACTACTCCAATTTTTTAATAATAACAAACACATCAACAAAAA
CACTACAAAATTTAACCACAACATGAACAACCTCTACAGCAACAACCCTCTCCATT
GCCCTCCTAATACTATCAACCAGCGGCCTACCACCATTCACAGGATTCATACCAAA
AATATTAATTCTTAATGAACTCATTTCACAAAAACTTATAACACTAGCAACCCTAGC
AATTATAACATCACTAGTTAGCTTATTATTTTACTTACGAATTACATACCTAATTATAA
TACTCACACCACCAATAACAACCCAATCATCAACAAAATGACGAACCCAAAACCA
AAAATCACAACTAATAACAATAATAACTCCAACAGCACTATTTATCACCCACCTAAT
CCCAGCAATCCCGTTCTAAAGAAGCTTAGGATT-AAGCTTATTAAACCAGTGGCCTT
CAACACCACAAACAAGGGAA-AACCCTTAGCTTCTGAAAGACCTATAGGACTTTCT
CCTACATCAT

32

GGCTGAACCATTATAACCATAGCCATCTCCCCAAACGTAGCAATCATAAATATCTCC
ATTTACATTATTATAACTACTCCAATTTTTTAATAATAACAAACACATCAACAAAAA
CACTACAAAATTTAACCACAACATGAACAACCTCTACAGCAACAACCCTCTCCATT
GCCCTCCTAATACTATCAACCAGCGGCCTACCACCATTCACAGGATTCATACCAAA
AATATTAATTCTTAATGAACTCATTTCACAAAAACTTACAACACTAGCAACCCTAGC
AATTATAACATCACTAGTTAGCTTATTATTTTACTTACGAATTACATACCTAATTATAA
TACTCACACCACCAATAACAACCCAATCATCAACAAAATGACGAACCCAAAACCA
AAAATCACAACTAATAACAATAATAACTCCAACAGCACTATTTATCACCCACCTAAT
CCCAGCAATCCCGTTCTAAAGAAGCTTAGGATT-AAGCTTATTAAACCAGTGGCCTT
CAACACCACAAACAAGGGAA-AACCCTTAGCTTCTGAAAGACCTATAGGACTTTCT
CCTACATCAT

33

GGCTGAACCATTATAACCATAGCCATCTCCCCAAACGTAGCAATCATAAATATCTCC
ATTTACATTATTATAACTACTCCAATTTTTTAATAATAACAAACACATCAACAAAAA
CACTACAAAATTTAACCACAACATGAACAACCTCTACAGCAACAACCCTCTCCATT
GCCCTCCTAATACTATCAACCAGCGGCCTACCACCATTCACAGGATTCATGCCAAA
AATATTAATTCTTAATGAACTCATTTCACAAAAACTTACAACACTAGCAACCCTAGC
AATTATAACATCACTAGTTAGCTTATTATTTTACTTACGAATTACATACCTAATTATAA
TACTCACACCACCAATAACAACCCAATCATCAACAAAATGACGAACCCAAAACCA
AAAATCACAACTAATAACAATAATAACTCCAACAGCACTATTTATCACCCACCTAAT
CCCAGCAATCCCGTTCTAAAGAAGCTTAGGATT-AAGCTTATTAAACCAGTGGCCTT
CAACACCACAAACAAGGGAA-AACCCTTAGCTTCTGAAAGACCTATAGGACTTTCT
CCTACATCAT

34

GGCTGAACCATTATAACCATAGCCATCTCCCCAAACGTAGCAATCATAAATATCTCC
ATTTACATTATTATAACTACTCCAATTTTTTTAATAATAACAAACACATCAACAAAAA
CACTACAAAATTTAACCACAACATGAACAACCTCTACAGCAACAACCCTCTCCATT
GCCCTCCTAATACTATCAACCAGCGGCCTACCCCCATTCACAGGATTCATACCAAA
AATATTAATTCTTAATGAACTCATTTCACAAAAACTTACAACACTAGCAACCCTAGC
AATTATAACATCACTAGTTAGCTTATTATTTTACTTACGAATTACATACCTAATTATAA
TACTCACACCACCAATAACAACCCAATCATCAACAAAATGACGAACCCAAAACCA
AAAATCACAACTAATAACAATAATAACTCCAACAGCACTATTTATCACCCACCTAAT
CCCAGCAATCCCGTTCTAAAGAAGCTTAGGATT-AAGCTTATTAAACCAGTGGCCTT
CAACACCACAAACAAGGGAA-AACCCTTAGCTTCTGAAAGACCTATAGGACTTTCT
CCTACATCAT

35

GGCTGAACCATTATAACCATAGCCATCTCCCCAAACGTAGCAATCATAAATATCTCC
ATTTACATTATTATAACTACTCCAATTTTTTTAATAATAACAAACACATCAACAAAAA
CACTACAAAATTTAACCACAACATGAACAACCTCTACAGCAACAACCCTCTCCATT
GCCCTCCTAATACTATCAACCAGCGGCCTACCACCATTCACAGGGTTCATACCAAA
AATACTAATTCTTAATGAACTCATTTCACAAAAACTTACAACACTAGCAACCCTAGC
AATTATAACATCACTAGTTAGCTTATTATTTTACTTACGAATTACATACCTAATTATAA
TACTCACACCACCAATAACAACCCAAGCATCAACAAAATGACGAACCCAAAACCA
AAAATCACAACTAATAACAATAATAACTCCAACAGCACTATTTATCACCCACCTAAT
CCCAGCAATCCCGTTCTAAAGAAGCTTAGGATT-AAACTTATTAAACCAGTGGCCTT
CAACACCACAAACAAGGGAA-AACCCTTAGCTTCTGAAAGACCTATAGGACTTTCT
CCTACATCAT

36

GGCTGAACCATTATAACCATAGCCATCTCCCCAAACGTAGCAATCATAAATATCTCC
ATTTACATTATTATAACTACTCCAATTTTTTTAATAATAACAAACACATCAACAAAAA
CACTACAAAATTTAACCACAACATGAACAACCTCTACAGCAACAACCCTCTCCATT
GCCCTCCTAATACTATCAACCAGCGGCCTACCACCATTCACAGGGTTCATACCAAA
AATACTAATTCTTAATGAACTCATTTCACAAAAACTTACAACACTAGCAACCCTAGC
AATTATAACATCACTAGTTAGCTTATTATTTTACTTACGAATTACATACCTAATTATAA
TACTCACACCACCAATAACAACCCAAGCATCAACAAAGTGACGAACCCAAAACCA
AAAATCACAACTAATAACAATAATAACTCCAACAGCACTATTTATCACCCACCTAAT
CCCAGCAATCCCGTTCTAAAGAAGCTTAGGATT-AAACTTATTAAACCAGTGGCCTT
CAACACCACAAACAAGGGAA-AACCCTTAGCTTCTGAAAGGCCTATAGGACTTTCT
CCTACATCAT

37

GGCTGAACCATTATAACCATAGCCATCTCCCCAAACGTAGCAATCATAAATATCTCC
ATTTACATTATTATAACTACTCCAATTTTTTTAATAATAACAAACACATCAACAAAAA
CACTACAAAATTTAACCACAACATGAACAACCTCTACAGCAACAACCCTCTCCATT
GCCCTCCTAATACTATCAACCAGCGGCCTACCACCATTCACAGGGTTCATACCAAA
AATACTAATTCTTAATGAACTCATTTCACAAAAACTTACAACACTAGCAACCCTAGC
AATTATAACATCACTAGTTAGCTTATTATTTTACTTACGAATTACATACCTAATTATAA
TACTCACACCACCAATAACAACCCAAGCATCAACAAAGTGACGAACCCAAAACCA
AAAATCACAACTAATAACAATAATAACTCCAACAGCACTATTTATCACCCACCTAAT

CCCAGCAATCCCGTCCTAAAGAAGCTTAGGATT-AAACTTATTAAACCAGTGGCCTT
CAACACCACAAACAAGGGAA-AACCCTTAGCTTCTGAAAGGCCTATAGGACTTTCT
CCTACATCAT
38
GGCTGAACCATTATAACAATAGCCATCTCCCCAAACGTAGCAATCATAAATATCTCC
ATTTACATTATTATAACTACTCCAATTTTTTTAATAATAACAAACACATCAACAAAAA
CACTACAAAATTTAACCACAACATGAACAACCTCTACAGCAACAACCCTCTCCATT
GCCCTCCTAATACTATCAACCAGCGGCCTACCACCATTCACAGGGTTCATACCAAA
AATACTAATTCTTAATGAACTCATTTCACAAAAACTTACAACACTAGCAACCCTAGC
AATTATAACATCACTAGTTAGCTTATTATTTTACTTACGAATTACATACCTAATTATAA
TACTCACACCACCAATAACAACCCAAGCATCAACAAAGTGACGAACCCAAAACCA
AAAATCACAACTAATAACAATAATAACTCCAACAGCACTATTTATCACCCACCTAAT
CCCAGCAATCCCGTTCTAAAGAAGCTTAGGATT-AAACTTATTAAACCAGTGGCCTT
CAACACCACAAACAAGGGAA-AACCCTTAGCTTCTGAAAGGCCTATAGGACTTTCT
CCTACATCAT
39
GGCTGAACCATTATAACCATAGCCATCTCCCCAAACGTAGCAATCATAAATATCTCC
ATTTACATTATTATAACTACTCCAATTTTTTTAATAATAACAAACACATCAACAAAAA
CACTACAAAATTTAACCACAACATGAACAACCTCTACAGCAACAACCCTCTCCATT
GCCCTCCTAATACTATCAACCAGCGGCCTACCACCATTCACAGGGTTCATACCAAA
AATACTAATTCTTAATGAACTCATTTCACAAAAACTTACAACACTAGCAACCCTAGC
AATTATAACATCACTAGTTAGCTTATTATTTTACTTACGAATTACATACCTAATTATAA
TACTCACACCACCAATAACAACCCAAGCATCAACAAAATGACGAACCCAAAACCA
AAAATCACAACTAATAACAATAATAACTCCAACAGCACTATTTATTACCCACCTAAT
CCCAGCAATCCCGTTCTAAAGAAGCTTAGGATT-AAACTTATTAAACCAGTGGCCTT
CAACACCACAAACAAGGGAA-AACCCTTAGCTTCTGAAAGACCTATAGGACTTTCT
CCTACATCAT
40
GGCTGAACCATTATAACCATAGCCATCTCCCCAAACGTAGCAATCATAAATATCTCC
ATTTACATTATTATAACTACTCCAATTTTTTTAATAATAACAAACACATCAACAAAAA
CACTACAAAATTTAACCACAACATGAACAACCTCTACAGCAACAACCCTCTCCATT
GCCCTCCTAATACTATCAACCAGCGGCCTACCACCATTCACAGGATTCATACCAAA
AATACTAATTCTTAATGAACTCATTTCACAAAAACTTACAACACTAGCAACCCTAGC
AATTATAACCTCACTAGTTAGCTTATTATTTTACTTACGAATTACATACCTAATTATAA
TACTCACACCACCAATAACAACCCAATCATCAACAAAATGACGAACCCAAAACCA
AAAATCACAACTAATAACAATAATAACTCCAACAGCACTATTTATCACCCACCTAAT
CCCAGCAATCCCGTTCTAAAGAAGCTTAGGATT-AAACTTATTAAACCAGTGGCCTT
CAACACCACAAACAAGGGAA-AACCCTTAGCTTCTGAAAGACCTATAGGACTTTCT
CCTACATCAT
41
GGCTGAACCATCATAACCATAGCCATCTCCCCAAACGTAGCAATCATAAATATCTCC
ATTTACATTATTATAACTACTCCAATTTTTTTAATAATAACAAACACATCAACAAAAA
CACTACAAAATTTAACCACAACATGAACAACCTCTACAGCAACAACCCTCTCCATT
GCCCTCCTAATACTATCAACCAGCGGCCTACCACCATTAACAGGATTTATACCAAAA

ATACTAATTCTTAATGAACTCATTTCACAAAAACTTACAACGCTAGCAACCCTAGCA
ATTATAACATCACTAATTAGCTTATTATTTTACTTACGAATCACATACCTAATTATAAT
ACTCACACCACCAATAACAACCCAATCATCAACAAAATGACGAACCCAAAACCAA
AAATCACAACTAATAACAATAATAACTCCAACAGCACTATTTATTACCCACCTAATC
CCAGCAATCCCGCTCTAAAGAAGCTTAGGATT-AAGCTTATTAAACCAGTGGCCTTC
AACACCACAAACAAGGGAA-AACCCTTAGCTTCTGAAAGACCTATAGGACTTTCTC
CTATATCAT
42
GGCTGAACCATCATAACCATAGCCATCTCCCCAAACGTAGCAATCATAAATATCTCC
ATTTACATTATTATAACTACTCCAATTTTTTAATAATAACAAACACATCAACAAAAA
CACTACAAAATTTAACCACAACATGAACAACCTCTACAGCAACAACCCTCTCCATT
GCCCTCCTAATACTATCAACCAGCGGCCTACCACCATTAACAGGATTTATACCAAAA
ATACTAATTCTTAATGAACTCATTTCACAAAAACTTACAACGCTAGCAACCCTAGCA
ATTATAACATCACTAATTAGCTTATTATTTTACTTACGAATCACATACCTAATTATAGT
ACTCACACCACCAATAACAACCCAATCATCAACAAAATGACGAACCCAAAACCAA
AAATCACAACTAATAACAATAATAACTCCAACAGCACTATTTATTACCCACCTAATC
CCAGCAATCCCGCTCTAAAGAAGCTTAGGATT-AAGCTTATTAAACCAGTGGCCTTC
AACACCACAAACAAGGGAA-AACCCTTAGCTTCTGAAAGACCTATAGGACTTTCTC
CTATATCAT
43
GGCTGAACCATTATAACCATAGCCCTCTCCCCAAACCTAGCAATCATAAATATCTCC
ATTTACATTATTATAACTACCCCAATTTTTTAATGATAACAAATACATCAACAAAAA
CACTACAAAATTTAACCACAACATGAACAACCTCTACAACAACAACCCTCTCCATT
GCCCTCCTAATATTATCAACCAGCGGCCTACCACCATTCACAGGATTTATACCAAAA
ATACTAATTCTTAATGAACTCATTTCACAAAAACTTACAACACTAGCAACCCTAGCA
ATTATAACATCACTAGTCAGCTTATTATTTTACTTACGAATCACGTACCTAATTATAAT
ACTTACATCACCAATAACAACCCCATCATCAACAAAATGACGAACCCAAAACCAA
AAGTCACAACTAATAACAATAATAACTCCAACAGCACTATTTATTACCCACCTAATC
CCAGCAATCCCGTTCTAAAGAAGCTTAGGATC-AAGCTTATCAAACCAGTGGCTTT
CAACTCCACAAACAAGGGTA-TACCCTTAGCTTCTGAAAGACCTATAGGATTTTCTC
CTACATCAT
44
GGCTGAACCATTATAACCATAGCCCTCTCCCCAAACCTAGCAATCATAAATATCTCC
ATTTACATTATTATAACTACCCCAATTTTTTAATGATAACAAATACATCAACAAAAA
CACTACAAAATTTAACCACAACATGAACAACCTCTACAACAACAACCCTCTCCATT
GCCCTCCTAATATTATCAACCAGCGGCCTACCACCATTCACAGGATTTATACCAAAA
ATACTAATTCTTAATGAACTCATTTCACAAAAACTTACAACACTAGCAACCCTAGCA
ATTATAACATCACTAGTCAGCTTATTATTTTACTTACGAATCACGTACCTAATTATAAT
ACTTACATCACCAATAACAACCCCATCATCAACAAAATGACGAACCCAAAACCAA
AAATCACAACTAATAACAATAATAACTCCAACAGCACTATTTATTACCCACCTAATC
CCAGCAATCCCGTTCTAAAGAAGCTTAGGATC-AAGCTTATCAAACCAGTGGCTTT
CAACTCCACAAACAAGGGTA-TACCCTTAGCTTCTGAAAGACCTATAGGATTTTCTC
CTACATCAT
45

GGCTGAACCATTATAATTATAGCCCTCTCCCCAAACTTAGCGATTATAAATATCTCCG
TTTACATTATGATAACCACCCCAATTTTTTAATAATAACAAGCACATCAACAAAAA
CACTACAAAATTTAACCACAACATGAACAACCTCTACAGCAACAGCCCTCTCCATT
GCCCTCCTAATACTATCAACCAGTGGCCTCCCACCGTTCACAGGATTTATACCAAA
AATTCTAGCTCTTAATGAACTTATTACACAAAAACTTACAACACTAGCAACCCTGG
CAATTATAACATCACTAATTAGCTTATTATTTTATTTACGAATCGCATATCTAATTATA
GTGCTCACATCACCAATAACGACCCCATCATCAACAAAATGACGAATCCAAAACC
AAAAACCACAACTAATAACAATAATAACCCCAACAGCACTATTCATTACCCACCTA
ATTCCAGCAATCCCGCTCTAAAGAAGCTTAGGATT-AA-CTTATCAAACCAGTGGCC
TTCAACACCACAAACAAGGGA-GAACCCTTAGCTTCTGAAAGACCTATAGGACTTT
CTCCTACATCAT
46
GGCTGAACCATTATAATTATAGCCCTCTCCCCAAACTTAGCGATTATAAATATCTCCG
TTTACATTATGATAACCACCCCAATTTTTTAATAATAACAAGCACATCAACAAAAA
CACTACAAAATTTAACCACAACATGAACAACCTCTACAGCAACAGCCCTCTCCATT
GCCCTCCTAATACTATCAACCAGTGGCCTCCCACCGTTCACAGGATTTATACCAAA
AATTCTAGCTCTTAATGAACTTATTACACAAAAACTTACAACACTAGCAACCCTGG
CAATTATAACATCACTAATTAGCTTATTATTTTATTTACGAATCGCATATCTAATTATA
GTGCTCACATCACCAATAACGACCCCATCATCAACAAAATGACGAATCCAAAACC
AAAAACCACAACTAATAACAATAATAACCCCAACAGCACTATTCATTACCCACCTA
ATCCCAGCAATCCCGCTCTAAAGAAGCTTAGGATT-AA-CTTATCAAACCAGTGGCC
TTCAACACCACAAACAAGGGA-GAACCCTTAGCTTCTGAAAGACCTATAGGACTTT
CTCCTACATCAT
47
GGCTGAACCATTATAATTATAGCCCTCTCCCCAAACTTAGCGATTATAAATATCTCCG
TTTACATTATGATAACCACCCCAATTTTTTAATAATAATAAGCACATCAACAAAAA
CACTACAAAATTTAACCACAACATGAACAACCTCTACAGCAACAGCCCTCTCCATT
GCCCTCCTAATACTATCAACCAGTGGCCTCCCACCGTTCACAGGATTTATACCAAA
AATTCTAGCTCTTAATGAACTTATTACACAAAAACTTACAACACTAGCAACCCTGG
CAATTATAACATCACTAATTAGCTTATTATTTTATTTACGAATCGCATATCTAATTATA
GTGCTCACATCACCAATAACGACCCCATCATCAACAAAATGACGAATCCAAAACC
AAAAACCACAACTAATAACAATAATAACCCCAACAGCACTATTCATTACCCACCTA
ATCCCAGCAATCCCGCTCTAAAGAAGCTTAGGATT-AA-CTTATCAAACCAGTGGCC
TTCAACACCACAAACAAGGGA-GAACCCTTAGCTTCTGAAAGACCTATAGGACTTT
CTCCTACATCAT
48
GGCTGAACCATTATAATTATAGCCCTCTCCCCAAACTTAGCGATTATAAATATTTCCG
TTTACATTATGATAACCACCCCAATTTTTTAATAATAACAAGCACATCAACAAAAA
CACTACAAAATTTAACCACAACATGAACAACCTCTACAGCAACAGCCCTCTCCATT
GCCCTCCTAATACTATCAACCAGTGGCCTCCCACCGTTCACAGGATTTATACCAAA
AATTCTAGCTCTTAATGAACTTATTACACAAAAACTTACAACACTAGCAACCCTGG
CAATTATAACATCACTAATTAGCTTATTATTTTATTTACGAATCGCATATCTAATTATA
GTGCTCACATCACCAATAACGACCCCATCATCAACAAAATGACGAATCCAAAACC
AAAAACCACAACTAATAACAATAATAACCCCAACAGCACTATTCATTACCCACCTA

ATCCCAGCAATCCCGCTCTAAAGAAGCTTAGGATT-AA-CTTATCAAACCAGTGGCC
TTCAACACCACAAACAAGGGA-GAACCCTTAGCTTCTGAAAGACCTATAGGACTTT
CTCCTACATCAT
49
GGCTGAACCATTATAATTATAGCCCTCTCCCCAAACTTAGCGATTATAAATATCTCCG
TTTACATTATGATAACCACCCCAATTTTTTAATAATAACAAGCACATCAACAAAAA
CACTACAAAATTTAACCACAACATGAACAACCTCTACAGCAACAGCCCTCTCCATT
GCCCTCCTAATACTATCAACCAGTGGCCTCCCACCATTCACAGGATTTGTACCAAA
AATTCTAGCTCTTAATGAACTTATTACACAAAAACTTACAACACTAGCAACCCTGG
CAATTATAACATCACTAATTAGCTTATTATTTTATTTACGAATCGCATATCTAATTATA
GTGCTCACATCACCAATAACGACCCCATCATCAACAAAATGACGAATCCAAAACC
AAAAACCACAACTAATAACAATAATAACCCCAACAGCACTATTCATTACCCACCTA
ATCCCAGCAATCCCGCTCTAAAGAAGCTTAGGATT-AA-CTTATCAAACCAGTGGCC
TTCAACACCACAAACAAGGGA-GAACCCTTAGCTTCTGAAAGACCTATAGGACTTT
CTCCTACATCAT
50
GGCTGAACCATTATAATTATAGCCCTCTCCCCAAACTTAGCAATTATAAATATCTCCG
TTTACATTATGATAACCACCCCAATTTTTTAATAATAACAAGCACATCAACAAAAA
CACTACAAAATTTAACCACAACATGAACAACCTCTACAGCAACAGCCCTCTCCATT
GCCCTCCTAATACTATCAACCAGTGGCCTCCCACCGTTCACAGGATTTATACCAAA
AATTCTAGCTCTTAATGAACTTATTACACAAAAACTTACAACACTAGCAACCCTGG
CAATTATAACATCACTAATTAGCTTATTATTTTATTTACGAATCGCATATCTAATTATA
GTGCTCACATCACCAATAACGACCCCATCATCAACAAAATGACGAATCCAAAACC
AAAAACCACAACTAATAACAATAATAACCCCAACAGCACTATTCATTACCCACCTA
ATCCCAGCAATCCCGCTCTAAAGAAGCTTAGGATT-AA-CTTATCAAACCAGTGGCC
TTCAACACCACAAACAAGGGA-GAACCCTTAGCTTCTGAAAGACCTATAGGACTTT
CTCCTACATCAT
51
GGCTGAACCATTATAATTATAGCCCTCTCCCCAAACTTAGCGATTATAAATATCTCCA
TTTACATTATGATAACCACCCCAATTTTTTAATAATAACAAGCACATCAACAAAAA
CACTACAAAATTTAACCACAACATGAACAACCTCTACAGCAACAGCCCTCTCCATT
GCCCTCCTAATACTATCAACCAGTGGCCTCCCACCGTTCACAGGATTTATACCAAA
AATTCTAGCTCTTAATGAACTTATTACACAAAAACTTACAACACTAGCAACCCTGG
CAATTATAACATCACTAATTAGCTTATTATTTTATTTACGAATCGCATATCTAATTATA
GTGCTCACATCACCAATAACGACCCCATCATCAACAAAATGACGAATCCAAAACC
AAAAACCACAACTAATAACAATAATAACCCCAACAGCACTATTCATTACCCACCTA
ATCCCAGCAATCCCGCTCTAAAGAAGCTTAGGATT-AA-CTTATCAAACCAGTGGCC
TTCAACACCACAAACAAGGGA-GAACCCTTAGCTTCTGAAAGACCTATAGGACTTT
CTCCTACATCAT
52
GGCTGAACCATTATAATTATAGCCCTCTCCCCAAACTTAGCGATTATAAATATCTCCG
TTTACATTATGATAACCACCCCAATTTTTTAATAATAACAAGCACATCAACAAAAA
CACTACAAAATTTAACCACAACATGAACAACCTCTACAGCAACAGCCCTCTCCATT
GCCCTCCTAATACTATCAACCAGTGGCCTCCCACCGTTCACAGGATTTATACCAAA

AATTCTAGCTCTTAATGAACTTATTACACAAAAACTTACAACACTAGCAACCCTGG
CAATTATAACATCACTAATTAGCTTATTATTTTATTTACGAATCGCATATCTAATTATA
GTGCTCACATCACCAATAACAACCCCATCATCAACAAAATGACGAATCCAAAACC
AAAAACCACAACTAATAACAATAATAACCCCAACAGCACTATTCATTACCCACCTA
ATCCCAGCAATCCCGCTCTAAAGAAGCTTAGGATT-AA-CTTATCAAACCAGTGGCC
TTCAACACCACAAACAAGGGA-GAACCCTTAGCTTCTGAAAGACCTATAGGACTTT
CTCCTACATCAT

53

GGCTGAACCATTATAATTATAGCCCTCTCCCCAAACTTAGCGATTATAAATATCTCCG
TTTACATTATGATAACCACCCCAATCTTTTTAATAATAACAAGCACATCAACAAAAA
CACTACAAAATTTAACCACAACATGAACAACCTCTACAGCAACAGCCCTCTCCATT
GCCCTCCTAATACTATCAACCAGTGGCCTCCCACCGTTCACAGGATTTATACCAAA
AATTCTAGCTCTTAATGAACTTATTACACAAAAACTTACAACACTAGCAACCCTGG
CAATTATAACATCACTAATTAGCTTATTATTTTATTTACGAATCGCATATCTAATTATA
GTGCTCACATCACCAATAACAACCCCATCATCAACAAAATGACGAATCCAAAACC
AAAAACCACAACTAATAACAATAATAACCCCAACAGCACTATTCATTACCCACCTA
ATCCCAGCAATCCCGCTCTAAAGAAGCTTAGGATT-AA-CTTATCAAACCAGTGGCC
TTCAACACCACAAACAAGGGA-GAACCCTTAGCTTCTGAAAGACCTATAGGACTTT
CTCCTACATCAT

54

GGCTGAACCATTATAATTATAGCCCTCTCCCCAAACTTAGCGATTATAAATATCTCCG
TTTACATTATGATAACCACCCCAATTTTTTTAATAATAACAAGCACATCAACAAAAA
CACTACAAAATTTAACCACAACATGAACAACCTCTACAGCAACAGCCCTCTCCATT
GCCCTCCTAATACTATCAACCAGCGGCCTCCCACCGTTCACAGGATTTATACCAAA
AATTCTAGCTCTTAATGAACTTATTACACAAAAACTTACAACACTAGCAACCCTGG
CAATTATAACATCACTAATTAGCTTATTATTTTATTTACGAATCGCATATCTAATTATA
GTGCTCACATCACCAATAACGACCCCATCATCAACAAAATGACGAATCCAAAACC
AAAAACCACAACTAATAACAATAATAACCCCAACAGCACTATTCATTACCCACCTA
ATCCCAGCAATCCCGCTCTAAAGAAGCTTAGGATT-AA-CTTATCAAACCAGTGGCC
TTCAACACCACAAACAAGGGA-GAACCCTTAGCTTCTGAAAGACCTATAGGACTTT
CTCCTACATCAT

55

GGCTGAACCATTATAATTATAGCCCTCTCCCCAAACTTAGCGATTATAAATATCTCCG
TTTACATTATGATAACCACCCCAATTTTTTTAATAATAACAAGCACATCAACAAAAA
CACTACAAAATTTAACCACAACATGAACAACCTCTACAGCAACAGCCCTCTCCATT
GCCCTCCTAATACTATCAACCAGTGGCCTCCCACCGTTCACAGGATTTATACCAAA
AATTCTAGCTCTTAATGAACTTATTACACAAAAACTTACAACACTAGCAACCCTGG
CAATTATAACATCACTAATTAGCTTATTATTTTATTTACGAATCGCATATCTAATTATA
GTACTCACATCACCAATAACGACCCCATCATCAACAAAATGACGAATCCAAAACCA
AAAACCACAACTAATAACAATAATAACCCCAACAGCACTATTCATTACCCACCTAAT
CCCAGCAATCCCGCTCTAAAGAAGCTTAGGATT-AA-CTTATCAAACCAGTGGCCTT
CAACACCACAAACAAGGGA-GAACCCTTAGCTTCTGAAAGACCTATAGGACTTTCT
CCTACATCAT

56

GGCTGAACCATTATAATTATAGCCCTCTCCCCAAACTTAGCGATTATAAATATCTCCG
TTTACATTATGATAACCACCCCAATTTTTTAATAATAACAAGCACATCAACAAAAA
CACTACAAAATTTAACCACAACATGAACAACCTCTACAGCAACAGCCCTCTCCATT
GCCCTCCTAATACTATCAACCAGTGGCCTCCCACCGTTCACAGGATTTATACCAAA
AATTCTAGCTCTTAATGAACTTATTACACAAAAACTTACAACACTAGCAACCCTGG
CAATTATAACATCACTAATTAGCTTATTATTTTATTTACGAATCGCATATCTAATTATA
GTGCTCACATCACCAATAACGACCCCATCATCAACAAAATGACGAATCCAAAACC
AAAAACCACAACTAATAACAATAATAACCCCAACAGCACTATTCATCACCCACCTA
ATCCCAGCAATCCCGCTCTAAAGAAGCTTAGGATT-AA-CTTATCAAACCAGTGGCC
TTCAACACCACAAACAAGGGA-GGACCCTTAGCTTCTGAAAGACCTATAGGACTTT
CTCCTACATCAT
57
GGCTGAACCATTATAATTATAGCCCTCTCCCCAAACTTAGCGATTATAAATATCTCCG
TTTACATTATGATAACCACCCCAATTTTTTAATAATAACAAGCACATCAACAAAAA
CACTACAAAATTTAACCACAACATGAACAACCTCTACAGCAACAGCCCTCTCCATT
GCCCTCCTAATACTATCAACCAGTGGCCTCCCACCGTTCACAGGATTTATACCAAA
AATTCTAGCTCTTAATGAACTTATTACACAAAAACTTACAACACTAGCAACCCTGG
CAATTATAACATCACTAATTAGCTTATTATTTTATTTACGAATCGCATATCTAATTATA
GTGCTCACATCACCAATAACGACCCCATCATCAACAAAATGACGAATCCAAAACC
AAAAACCACAACTAATAACAATAATAACCCCAACAGCACTATTCATCACCCACCTA
ATCCCAGCAATCCCGCTCTAAAGAAGCTTAGGATT-AA-CTTATTAAACCAGTGGCC
TTCAACACCACAAACAAGGGA-GAACCCTTAGCTTCTGAAAGACCTATAGGACTTT
CTCCTACATCAT
58
GGCTGAACCATTATAATTATAGCCCTCTCCCCAAACTTAGCGATTATAAATATCTCCG
TTTACATTATGATAACCACCCCAATTTTTTAATAATAACAAGCACATCAACAAAAA
CACTACAAAATTTAACCACAACATGAACAACCTCTACAGCAACAACCCTCTCCATT
GCCCTCCTAATACTATCAACCAGTGGCCTCCCACCGTTCACAGGATTTATACCAAA
AATTCTAGCTCTTAATGAACTTATTACACAAAAACTTACAACACTAGCAACCCTGG
CAATTATAACATCACTAATTAGCTTATTATTTTATTTACGAATCGCATATCTAATTATA
GTGCTCACATCACCAATAACGACCCCATCATCAACAAAATGACGAATCCAAAACC
AAAAACCACAACTAATAACAATAATAACTCCAACAGCACTATTCATTACCCACCTA
ATCCCAGCAATCCCGCTCTAAAGAAGCTTAGGATT-AA-CTTATCAAACCAGTGGCC
TTCAACACCACAAACAAGGGA-GAACCCTTAGCTTCTGAAAGACCTATAGGACTTT
CTCCTACATCAT
59
GGCTGAACCATTATAATTATAGCCCTCTCCCCAAACTTAGCGATTATAAATATCTCCG
TTTACATTATGATAACCACCCCAATTTTTTAATAATAACAAGCACATCAACAAAAA
CACTACAAAATTTAACCACAACATGAACAACCTCTACAGCAACAACCCTCTCCATT
GCCCTCCTAATACTATCAACCAGTGGCCTCCCACCGTTCACAGGATTTATACCAAA
AATTCTAGCTCTTAATGAACTTATTACACAAAAACTTACAACACTAGCAACCCTGG
CAATTATAACATCACTAATTAGCTTATTATTTTATTTACGAATCGCATATCTAATTATA
GTGCTCACATCACCAATAACGACCCCATCATCAACAAAATGACGAATCCAAAACC
AAAAACCACAACTAATAACAATAATAACTCCAACAGCACTATTCATTACCCACCTA

ATCCCAGCAATCCCGCTCTAAAGAAGCTTAGGATT-AA-CTTATCAAACCAGTGGCC
TTCAACACCACAAACAAGGGA-GGACCCTTAGCTTCTGAAAGACCTATAGGACTTT
CTCCTACATCAT

60

GGCTGAACCATTATAATTATAGCCCTCTCCCCAAACTTAGCGATTATAAATATCTCCG
TTTACATTATGATAACCACCCCAATTTTTTAATAATAACAAGCACATCAACAAAAA
CACTACAAAATTTAACCACAACATGAACAACCTCTACAGCAACAACCCTCTCCATT
GCCCTCCTAATACTATCAACCAGTGGCCTCCCACCGTTCACAGGATTTATACCAAA
AATTTAGCTCTTAATGAACTTATTACACAAAAACTTACAACACTAGCAACCCTGG
CAATTATAACATCACTAATTAGCTTATTATTTATTTACGAATCGCATATCTAATTATA
GTGCTCACATCACCAATAACGACCCCATCATCAACAAAATGACGAATCCAAAACC
AAAAACCACAACTAATAACAATAATAACTCCAACAGCACTATTCATTACCCACCTA
ATCCCAGCAATCCCGCTCTAAAGAAGCTTAGGATT-AA-CTTATCAAACCAGTGGCC
TTCAACACCACAAACAAGGGA-GGACCCTTAGCTTCTGAAAGACCTATAGGACTTT
CTCCTACATCAT

61

GGCTGAACCATTATAATTATAGCCCTCTCCCCAAACTTAGCGATTATAAATATCTCCG
TTTACATTATGATAACCACCCCAATTTTTTAATAATAACAAGCACATCAACAAAAA
CACTACAAAATTTAACCACAACATGAACAACCTCTACAGCAACAACCCTCTCCATT
GCCCTCCTAATACTATCAACCGGTGGCCTCCCACCGTTCACAGGATTTATACCAAA
AATTCTAGCTCTTAATGAACTTATTACACAAAAACTTACAACACTAGCAACCCTGG
CAATTATAACATCACTAATTAGCTTATTATTTATTTACGAATCGCATATCTAATTATA
GTGCTCACATCACCAATAACGACCCCATCATCAACAAAATGACGAATCCAAAACC
AAAAACCACAACTAATAACAATAATAACTCCAACAGCACTATTCATTACCCACCTA
ATCCCAGCAATCCCGCTCTAAAGAAGCTTAGGATT-AA-CTTATTAAACCAGTGGCC
TTCAACACCACAAACAAGGGA-GAACCCTTAGCTTCTGAAAGACCTATAGGACTTT
CTCCTACATCAT

62

GGCTGAACCATTATAATTATAGCCCTCTCCCCAAACTTAGCGATTATAAATATCTCCG
TTTACATTATGATAACCACCCCAATTTTTTAATAATAACAAGCACATCAACAAAAA
CACTACAAAATTTAACCACAACATGAACAACCTCTACAGCAACAACCCTCTCCATT
GCCCTCCTAATACTATCAACCAGTGGCCTCCCACCGTTCACAGGATTTATACCAAA
AATTCTAGCTCTTAATGAACTTATTACACAAAAACTTACAACACTAGCAACCCTGG
CAATTATAACATCACTAATTAGCTTATTATTTATTTACGAATCGCATATCTAATTATA
GTACTCACATCACCAATAACGACCCCATCATCAACAAAATGACGAATCCAAAACCA
AAAACCACAACTAATAACAATAATAACTCCAACAGCACTATTCATTACCCACCTAAT
CCCAGCAATCCCGCTCTAAAGAAGCTTAGGATT-AA-CTTATCAAACCAGTGGCCTT
CAACACCACAAACAAGGGA-GAACCCTTAGCTTCTGAAAGACCTATAGGACTTTCT
CCTACATCAT

63

GGCTGAACCATTATAATTATAGCCCTCTCCCCAAACTTAGCGATTATAAATATCTCCG
TTTACATTATGATAACCACCCCAATTTTTTAATAATAACAAGCACATCAACAAAAA
CACTACAAAATTTAACCACAACATGAACAACCTCTACAGCAACAACCCTCTCCATT
GCCCTCCTAATACTATCAACCAGTGGCCTCCCACCGTTCACAGGATTTATACCAAA

AATTCTAGCTCTTAATGAACTTATTACACAAAAACTTACAACACTAGCAACCCTGG
CAATTATAACATCACTAATTAGCTTATTATTTATTTACGAATCGCATATCTAATTATA
GTCCTCACATCACCAATAACGACCCCATCATCAACAAAATGACGAATCCAAAACCA
AAAACCACAACTAATAACAATAATAACTCCAACAGCACTATTCATTACCCACCTAAT
CCCAGCAATCCCGCTCTAAAGAAGCTTAGGATT-AA-CTTATCAAACCAGTGGCCTT
CAACACCACAAACAAGGGA-GAACCCTTAGCTTCTGAAAGACCTATAGGACTTTCT
CCTACATCAT
64
GGCTGAACCATTATAATTATAGCCCTCTCCCCAAACTTAGCGATTATAAATATCTCCG
TTTACATTATGATAACCACCCCAATTTTTTAATAATAACAAGCACATCAACAAAAA
CACTACAAAATTTAACCACAACATGAACAACCTCTACAGCAACAACCCTCTCCATT
GCCCTCCTAATACTATCAACCAGCGGCCTCCCACCGTTCACAGGATTTATACCAAA
AATTCTAGCTCTTAATGAACTTATTACACAAAAACTTACAACACTAGCAACCCTAG
CAATTATAACATCACTAATTAGCTTATTATTTATTTACGAATCGCATATCTAATTATA
GTGCTCACATCACCAATAACGACCCCATCATCAACAAAATGACGAATCCAAAACC
AAAAACCACAACTAATAACAATAATAACTCCAACAGCACTATTCATTACCCACCTA
ATCCCAGCAATCCCGCTCTAAAGAAGCTTAGGATT-AA-CTTATCAAACCAGTGGCC
TTCAACACCACAAACAAGGGA-GAACCCTTAGCTTCTGAAAGACCTATAGGACTTT
CTCCTACATCAT
65
GGCTGAACCATTATAATTATAGCCCTCTCCCCAAACTTAGCGATTATAAATATCTCCG
TTTACATTATGATAACCACCCCAATTTTTTAATAATAACAAGCACATCAACAAAAA
CACTACAAAATTTAACCACAACATGAACAACCTCTACAGCAACAACCCTCTCCATT
GCCCTCCTAATACTATCAACCAGTGGCCTCCCACCGTTCACAGGATTTATACCAAA
AATTCTAGCTCTTAATGAACTTATTACACAAAAACTTACAACACTAGCAACCCTAG
CAATTATAACATCACTAATTAGCTTATTATTTATTTACGAATCACATATCTAATTATAA
TGCTCACATCACCAATAACGACCTCATCATCAACAAAATGACGAATCCAAAACCAA
AAACCACAACTAATAACAATAATAACTCCAACAGCACTATTCATTACCCACCTAATC
CCAGCAATCCCGCTCTAAAGAAGCTTAGGATT-AA-CTTATCAAACCAGTGGCCTTC
AACACCACAAACAAGGGA-GAACCCTTAGCTTCTGAAAGACCTATAGGACTTTCTC
CTACATCAT
66
GGCTGAACCATTATAATTATAGCCCTCTCCCCAAACTTAGCGATTATAAATATCTCCG
TTTACATTATGATAACCACCCCAATTTTTTAATAATAACAAGCACATCAACAAAAA
CACTACAAAATTTAACCACAACATGAACAACCTCTACAGCAACAACCCTCTCCATT
GCCCTCCTAATACTATCAACCAGTGGCCTCCCACCGTTCACAGGATTTATACCAAA
AATTCTAGCTCTTAATGAACTTATTACACAAAAACTTACAACACTAGCAACCCTAG
CAATTATAACATCACTAATTAGCTTATTATTTATTTACGAATCACATATCTAATTATAA
TGCTCACATCACCAATAACGACCCCATCATCAACAAAATGACGAATCCAAAACCA
AAAACCACAACTAATAACAATAATAACTCCAACAGCACTATTCATTACCCACCTAAT
CCCAGCAATCCCGCTCTAAAGAAGCTTAGGATT-AA-CTTATCAAACCAGTGGCCTT
CAACACCACAAACAAGGGA-GAACCCTTAGCTTCTGAAAGACCTATAGGACTTTCT
CCTACATCAT
67

GGCTGAACCATTATAATTATAGCCCTCTCCCCAAACTTAGCGATTATAAATATCTCCG
TTTACATTATGATAACCACCCCAATTTTTTAATAATAACAAGCACATCAACAAAAA
CACTACAAAATTTAACCACAACATGAACAACCTCTACAGCAACAACCCTCTCCATT
GCCCTCCTAATACTATCAACCAGTGGCCTCCCACCGTTCACAGGATTTATACCAAA
AATTCTAGCTCTTAATGAACTTATTACACAAAAACTTACAACACTAGCAACCCTAG
CAATTATAACATCACTAATTAGCTTATTATTTTATTTACGAATCGCATATCTAATTATAA
TGCTCACATCACCAATAACGACCCCATCATCAACAAAATGACGAATCCAAAACCA
AAAACCACAACTAATAACAATAATAACTCCAACAGCACTATTCATTACCCACCTAAT
CCCAGCAATCCCGCTCTAAAGAAGCTTAGGATT-AA-CTTATCAAACCAGTGGCCTT
CAACACCACAAACAAGGGA-GAACCCTTAGCTTCTGAAAGACCTATAGGACTTTCT
CCTACATCAT

68

GGCTGAACCATTATAATTATAGCCCTCTCCCCAAACTTAGCGATTATAAATATCTCCG
TTTACATTATGATAACCACCCCAATTTTTTAATAATAACAAGCACATCAACAAAAA
CACTACAAAATTTAACCACAACATGAACAACCTCTACAGCAACAACCCTCTCCATT
GCCCTCCTAATACTATCAACCAGTGGCCTCCCACCGTTCACAGGATTTATACCAAA
AATTCTAGCTCTTAATGAACTTATTACACAAAAACTTACAACACCAGCAACCCTAG
CAATTATAACATCACTAATTAGCTTATTATTTTATTTACGAATCGCATATCTAATTATAA
TGCTCACATCACCAATAACGACCCCATCATCAACAAAATGACGAATCCAAAACCA
AAAACCACAACTAATAACAATAATAACTCCAACAGCACTATTCATTACCCACCTAAT
CCCAGCAATCCCGCTCTAAAGAAGCTTAGGATT-AA-CTTATCAAACCAGTGGCCTT
CAACACCACAAACAAGGGA-GAACCCTTAGCTTCTGAAAGACCTATAGGACTTTCT
CCTACATCAT

69

GGCTGAACCATTATAATTATAGCCCTCTCCCCAAACTTAGCGATTATAAATATCTCCG
TTTACATTATGATAACCACCCCAATTTTTTAATAATAACAAGCACATCAACAAAAA
CACTACAAAATTTAACCACAACATGAACAACCTCTACAGCAACAACCCTCTCCATT
GCCCTCCTAATACTATCAACCAGTGGCCTCCCACCGTTCACAGGATTTATACCAAA
AATTCTAGCTCTTAATGAACTTATTACACAAAAACTTACAACACTAGCAACCCTAG
CAATTATAACATCACTAATTAGCTTATTATTTTATTTACGAATCGCATATCTAATTATAA
TGCTCACATCACCAATAACGACCCCGTCATCAACAAAATGACGAATCCAAAACCA
AAAACCACAACTAATAACAATAATAACTCCAACAGCACTATTCATTACCCACCTAAT
CCCAGCAATCCCGCTCTAAAGAAGCTTAGGATT-AA-CTTATCAAACCAGTGGCCTT
CAACACCACAAACAAGGGA-GAACCCTTAGCTTCTGAAAGACCTATAGGACTTTCT
CCTACATCAT

70

GGCTGAACCATTATAATTATAGCCCTCTCCCCAAACTTAGCGATTATAAATATCTCCG
TTTACATTATGATAACCACCCCAATTTTTTAATAATAACAAGCACATCAACAAAAA
CACTACAAAATTTAACCACAACATGAACAACCTCTACAGCAACAACCCTCTCCATT
GCCCTCCTAATACTATCAACCAGTGGCCTCCCACCGTTCACAGGATTTATACCAAA
AATTCTAGCTCTTAATGAACTTATTACACAAAAACTTACAACACTAGCAACCCTAG
CAATTATAACATCACTAATTAGCTTATTATTTTATTTACGAATCGCATATCTAATTATAA
TGCTCACATCACCAATAACAACCCCGTCATCAACAAAATGACGAATCCAAAACCA
AAAACCACAACTAATAACAATAATAACTCCAACAGCACTATTCATTACCCACCTAAT

CCCAGCAATCCCGCTCTAAAGAAGCTTAGGATT-AA-CTTATCAAACCAGTGGCCTT
CAACACCACAAACAAGGGA-GAACCCTTAGCTTCTGAAAGACCTATAGGACTTTCT
CCTACATCAT

71

GGCTGAACCATTATAATTATAGCCCTCTCCCCAAACTTAGCAATTATAAATATCTCCA
TTTACATTATGATAACTACCCCAATTTTTTAATAATAACAAGCACATCAACAAAAA
CACTACAAAATTTAACCACAACATGAACAACCTCTACAGCAACAACCCTCTCCCTT
GCCCTCCTAATACTATCAACCAGCGGTCTCCCACCGTTCACAGGATTTATACCAAA
AATTCTAGCTCTTAATGAACTTATTACACAAAAACTTACAACACTAGCAACCCTAG
CAATTATAACATCGCTAGTTAGCTTATTATTTTATTTACGAATCGCATATCTAATTATA
ATACTCACATCACCAATAACAACCACATCATCAACAAAATGACGAACCCAAAACC
AAAAACCACAACTAATAACAATAATAACTCCAACAGCACTATTCATTACCCACCTA
ATCCCAGCAATCCCGCTCTAAAGAAGCTTAGGATT-AAGCTTATCAAACCAGTGGC
CTTCAACACCACAAACAAGGGATAAACCCTTAGCTTCTGAAAGACCTATAGGACTT
TCTCCTACATCAT

72

GGCTGAACCATTATAATTATAGCCCTCTCCCCAAACTTAGCAATTATAAATATCTCCA
TTTACATTATGATAACTACCCCAATTTTTTAATAATAACAAACACATCAACAAAAA
CACTACAAAATTTAACCACAACATGAACAACCTCTACAGCAACAACCCTCTCCCTT
GCCCTCCTAATACTATCAACCAGCGGTCTCCCACCGTTCACAGGATTTATACCAAA
AATTCTAGCTCTTAATGAACTTATTACACAAAAACTTACAACACTAGCAACCCTAG
CAATTATAACATCGCTAGTTAGCTTATTATTTTATTTACGAATCGCATATCTAATTATA
ATACTCACATCACCAATAACAACCACATCATCAACAAAATGACGAACCCAAAACC
AAAAACCACAACTAATAACAATAATAACTCCAACAGCACTATTCATTACCCACCTA
ATCCCAGCAATCCCGCTCTAAAGAAGCTTAGGATT-AAGCTTATCAAACCAGTGGC
CTTCAACACCACAAACAAGGGATAAACCCTTAGCTTCTGAAAGACCTATAGGACTT
TCTCCTACATCAT

73

GGCTGAACCATTATAATTATAGCCCTCTCCCCAAACTTAGCAATTATAAATATCTCCA
TTTACACTATGATAATTACCCCAATTTTTTAATAATAACAGACACATCAACAAAAA
CACTACAAAATTTAACCACAACATGAACAACCTCTACAGCAACAACCCTCTCCCTT
GCCCTCCTAATACTATCAACCAGCGGTCTCCCACCGTTCACAGGATTTATACCAAA
AATTCTAGCTCTTAATGAACTTATCACACAAAAACTTACAACACTAGCAACCCTAG
CAATTATAACATCGCTAGTTAGCTTATTATTTTACTTACGAATCACATATCTAATTATA
ATACTCACATCACCAATAACAACCCCATCATCAACAAAATGACGAACCCAAAACCA
AAAACCACAACTAATAACAATAATAACTCCAACAGCACTATTCATTACCCACCTAAT
CCCAGCAATCCCACTCTAAAGAAGCTTAGGATT-AAGCTTATCAAACCAGTGGCCT
TCAACACCACAAACAAGGGATAAACCCTTAGCTTCTGAAAGACCTATAGGACTTTC
TCCTACATCAT

74

GGCTGAACCATTATAATTATAGCCCTTTCCCCAAATTTAGCAATTATAAATATCTCCA
CTTACATTATTATAACCACCCCAATTTTTCTAATAATAACAAATACATCAACAAAAAC
ACTACAAAATTTAACCACAACATGAACAACCTCTACAGCAACAACCCTCTCCATTG
CCCTCCTAATGCTATCAACCAGCGGCCTCCCACCATTCACAGGGTTTATACCAAAA

ATGCTAGCTCTTAATGAACTTATTACACAAAAACTTACAACGCTAGCAACCCTAGC
AGTTATAACATCATTAATTAGCTTATTATTTTACTTACGAATCGCATATTTAATTATAAT
GCTCACATCTCCAATAACAACCCCATCATCAACAAAATGACGAACCAAAAACCAA
AAACCACAACTAATAACAATAATAACTCCAACAGCACTATTCATCACCCACCTAATC
CCAGCAATCCCCCTCTAAAGAAGCTTAGGATT-AAACTTATTAAACCAGTGGCCTTC
AACACCACAAACAAGGGA-AAACCCTTAGCTTCTGAAAGACCCATAGGACTCTCT
CCTACATCAT
75
GGCTGAACCATTATAATTATAGCCCTTTCCCCAAATTTAGCAATTATAAATATCTCCA
CTTACATTATTATAACCACCCCAATTTTTCTAATAATAACAAATACATCAACAAAAAC
ACTACAAAATTTAACCACAACATGAACAACCTCTACAGCAACAACCCTCTCCATTG
CCCTCCTAATGCTATCAACCAGCGGCCTCCCACCATTCACAGGGTTTATACCAAAA
ATGCTAGCTCTTAATGAACTTATTACACAAAAACTTACAACGCTAGCAACCCTCGC
AGTTATAACATCATTAATTAGCTTATTATTTTACTTACGAATCGCATATTTAATTATAAT
GCTCACATCTCCAATAACAACCCCATCATCAACAAAATGACGAACCAAAAACCAA
AAACCACAACTAATAACAATAATAACTCCAACAGCACTATTCATCACCCACCTAATC
CCAGCAATCCCCCTCTAAAGAAGCTTAGGATT-AAACTTATTAAACCAGTGGCCTTC
AACACCACAAACAAGGGA-AAACCCTTAGCTTCTGAAAGACCCATAGGACTCTCT
CCTACATCAT
76
GGCTGAACCATTATAATTATAGCCCTTTCCCCAAATTTAGCAATTATAAACATCTCCA
CTTACATTATTATAACCACCCCAATTTTTCTAATAATAACAAATACATCAACAAAAAC
ACTACAAAATTTAACCACAACATGAACAACCTCTACAGCAACAACCCTCTCCATTG
CCCTCCTAATGCTATCAACCAACGGCCTCCCACCATTCACAGGGTTTATACCAAAA
ATGCTAGCTCTTAATGAACTTATTACACAAAAACTTACAACGCTAGCAACCCTAGC
AGTTATAACATCATTAATTAGCTTATTATTTTACTTACGAATCGCATATTTAATTATAG
TGCTCACATCGCCAATAACAACCCCATCATCAACAAAATGACGAACCAAAAACCA
AAAACCACAACTAATAACAATAATAACTCCAACAGCACTGTTCATCACCCACCTAA
TCCCAGCAATCCCCTTCTAAAGAAGCTTAGGATT-AAACTTATTAAACCAGTGGCCT
TCAACACCACAAACAAGGGA-AAACCCTTAGCTTCTGAAAGACCCATAGGACTCT
CTCCTACATCAT
77
GGCTGAACCATTATAATTATAGCCCTTTCCCCAAATTTAGCAATTATAAACATCTCCA
CTTACATTATTATAACCACCCCAATTTTTCTAATAATAACAAATACATCAACAAAAAC
ACTACAAAATTTAACCACAACATGAACAACCTCTACAGCAACAACCCTCTCCATCG
CCCTCCTAATGCTATCAACCAACGGCCTCCCACCATTCACAGGGTTTATACCAAAA
ATGCTAGCTCTTAATGAACTTATTACACAAAAACTTACAACGCTAGCAACCCTAGC
AGTTATAACATCATTAATTAGCTTATTATTTTACTTACGAATCGCATATTTAATTATAG
TGCTCACATCGCCAATAACAACCCCATCATCAACAAAATGACGAACCAAAAACCA
AAAACCACAACTAATAACAATAATAACTCCAACAGCACTGTTCATCACCCACCTAA
TCCCAGCAATCCCCTTCTAAAGAAGCTTAGGATT-AAACTTATTAAACCAGTGGCCT
TCAACACCACAAACAAGGGA-AAACCCTTAGCTTCTGAAAGACCCATAGGACTCT
CTCCTACATCAT
78

GGCTGAACCATTATAATTATAGCCCTTTCCCCAAATTTAGCAATTATAAACATCTCCA
CTTACATTATTATAACCACCCCAATTTTTCTAATAATAACAAATACATCAACAAAAAC
ACTACAAAATTTAACCACAACATGAACAACCTCTACAGCAACAACCCTCTCCATTG
CCCTCCTAATGCTATCAACCAGCGGCCTCCCACCATTCACAGGGTTTATACCAAAA
ATGCTAGCTCTTAATGAACTTATTACACAAAAACTTACAACGCTAGCAACCCTAGC
AGTTATAACATCATTAATTAGCTTATTATTTTACTTACGAATCGCATATTTAATTATAG
TGCTCACATCGCCAATAACAACCCCATCATCAACAAAATGACGAACCAAAAACCA
AAAACCACAACTAATAACAATAATAACTCCAACAGCACTATTCATCACCCACCTAA
TCCCAGCAATCCCCTTCTAAAGAAGCTTAGGATT-AAACTTATTAAACCAGTGGCCT
TCAACACCACAAACAAGGGA-AAACCCTTAGCTTCTGAAAGACCCATAGGACTCT
CTCCTACATCAT
79
GGCTGAACCATTATAATTATAGCCCTTTCCCCAAATTTAGCAATTATAAACATCTCCA
CTTACATTATTATAACCACCCCAATTTTTCTAATAATAACAAATACATCAACAAAAAC
ACTACAAAATTTAACCACAACATGAACAACCTCTACAGCAACAACCCTCTCCATTG
CCCTCCTAATGCTATCAACCAGCGGCCTCCCACCATTCACAGGGTTTATACCAAAA
ATGCTAGCTCTTAATGAACTTATTACACAAAAACTCACAACGCTAGCAACCCTAGC
AGTTATAACATCATTAATTAGCTTATTATTTTACTTACGAATCGCATATTTAATTATAG
TGCTCACATCGCCAATAACAACCCCATCATCAACAAAATGACGAACCAAAAACCA
AAAACCACAACTAATAACAATAATAACTCCAACAGCACTATTCATCACCCACCTAA
TCCCAGCAATCCCCTTCTAAAGAAGCTTAGGATT-AAACTTATTAAACCAGTGGCCT
TCAACACCACAAACAAGGGA-AAACCCTTAGCTTCTGAAAGACCCATAGGACTCT
CTCCTACATCAT
80
GGCTGAACCATTATAATTATAGCCCTTTCCCCAAATTTAGCAATTATAAACATCTCCA
CTTACATTATTATAACCACCCCAATTTTTCTAATAATAACAAATACATCAACAAAAAC
ACTACAAAATTTAACCACAACATGAACAACCTCTACAGCAACAACCCTCTCCATTG
CCCTCCTAATGCTATCAACCAGCGGCCTCCCACCATTTACAGGGTTTATACCAAAAA
TGCTAGCTCTTAATGAACTTATTACACAAAAACTTACAACGCTAGCAACCCTAGCA
GTTATAACATCATTAATTAGCTTATTATTTTACTTACGAATCGCATATTTAATTATAGT
GCTCACATCGCCAATAACAACCCCATCATCAACAAAATGACGAACCAAAAACCAA
AAACCACAACTAATAACAATAATAACTCCAACAGCACTATTCATCACCCACCTAATC
CCAGCAATCCCCTTCTAAAGAAGCTTAGGATT-AAACTTATTAAACCAGTGGCCTTC
AACACCACAAACAAGGGA-AAACCCTTAGCTTCTGAAAGACCCATAGGACTCTCT
CCTACATCAT
81
GGCTGAACCATTATAATTATAGCCCTTTCCCCAAATTTAGCAATTATAAACATCTCCA
CTTACATTGTTATAACCACCCCAATTTTTCTAATAATAACAAATACATCAACAAAAA
CACTACAAAATTTAACCACAACATGAACAACCTCTACAGCAACAACCCTCTCCATT
GCCCTCCTAATGCTATCAACCAGCGGCCTCCCACCATTTACAGGGTTTATACCAAA
AATGCTAGCTCTTAATGAACTTATTACACAAAAACTTACAACGCTAGCAACCCTAG
CAGTTATAACATCATTAATTAGCTTATTATTTTACTTACGAATCGCATATTTAATTATA
GTGCTCACATCGCCAATAACAACCCCATCATCAACAAAATGACGAACCAAAAACC
AAAAACCACAACTAATAACAATAATAACTCCAACAGCACTATTCATCACCCACCTA

ATCCCAGCAATCCCCTTCTAAAGAAGCTTAGGATT-AAACTTATTAAACCAGTGGCC
TTCAACACCACAAACAAGGGA-AAACCCTTAGCTTCTGAAAGACCCATAGGACTC
TCTCCTACATCAT
82
GGCTGAACCATTATAATTATAGCCCTTTCCCCAAATTTAGCAATTATAAACATCTCCA
CTTACATTATTATAACCACCCCAATTTTTCTAATAATAACAAATACATCAACAAAAAC
ACTACAAAATTTAACCACAACATGAACAACCTCTACAGCAACAACCCTCTCCATTG
CCCTCCTAATGCTATCAACCAGCGGCCTCCCACCATTTACAGGGTTTATACCAAAAA
TGCTAGCTCTTAATGAACTTATTACACAAAAACTTACAACGCTAGCAACCCTAGCA
GTTATAACATCATTAATTAGCTTATTATTTTACTTACGAATCGCATATTTAATTATAGT
GCTCACATCGCCAATAACAACCCCATCATCAACAAAATGACGAACCAAAAACCAA
AAACCACAACTAATAACAATAATAACTCCAACAGCACTATTCATCACCCACCTAATC
CCAGCAATCCCCTTCTAAAGAAGCTTAGGATT-AAACTTATCAAACCAGTGGCCTTC
AACACCACAAACAAGGGA-AAACCCTTAGCTTCTGAAAGACCCATAGGACTCTCT
CCTACATCAT
83
GGCTGAACCATTATAATTATAGCCCTTTCCCCAAATTTAGCAATTATAAACATCTCCA
CTTACATTATTATAACCACCCCAATTTTTCTAATAATAACAAATACATCAACAAAAAC
ACTACAAAATTTAACCACAACATGAACAACCTCTACAGCAACAACCCTCTCCATTG
CCCTCCTAATGCTATCAACCAGCGGCCTCCCACCATTCACAGGATTTATACCAAAAA
TGCTAGCTCTTAATGAACTTATTACACAAAAACTTACAACGCTAGCAACCCTAGCA
GTTATAACATCATTAATTAGCTTATTATTTTACTTACGAATCGCATATTTAATTATAGT
GCTCACATCGCCAATAACAACCCCATCATCAACAAAATGACGAACCAAAAACCAA
AAACCACAACTAATAACAATAATAACTCCAACAGCACTATTCATCACCCACCTAATC
CCAGCAATCCCCTTCTAAAGAAGCTTAGGATT-AAACTTATTAAACCAGTGGCCTTC
AACACCACAAACAAGGGA-AAACCCTTAGCTTCTGAAAGACCCATAGGACTCTCT
CCTACATCAT

The 8 AME genotype sequences (aligned) detected in *P. theobaldi*

AME_Hap_1
GGGGGAGATGTGAAATCGGTGTGCATGATGCACCAAAGTCCCTTTCAAGCTCTCCATC
CAATGCCACCACCTCTCCACCAGGTACAGCAACAGCCACCTCTGAACCCACACATGCA
GCTACCTGGACATAACACATTTGTGCCAATGACTGGACAGAATACATTAGTGCCACAGT
ATCAACCAGCTCACGCAGGTCCAGTTCACCAGCCAGTTCCACCAGTTGCAGGAGAGC
CACCAATGCACCCTCAGCCACCAGCACATCCAAATCAGCCAATGCACCCTCAGCTACC
AAACCCACCAATGTACCCAGTGCAGCCACTGCCTCCACTGATCCCAGATAGACCTCTT
GAGTCATGGCCAGTACCTGATAA

AME_Hap_2
GGGGGAGATGTGAAATCGGTGTGCATGATGCACCAAAGTCCCTTTCAAGCTCTCCATC
CAATGCCACCACCTCTCCACCAGGTACAGCAACAGCCACCTCTGAACCCACACATGCA
GCTACCTGGACATAACACATTTGTGCCAATGACTGGACAGAATACATTAGTGCCACAGT
ATCAACCAGCTCACGCAGGTCCAGTTCACCAGCCAGTTCCACCAGTTGCAGGAGAGC
CACCAATGCACCCTCAGCCACCAGCACATCCAAATCAGCCAATCCACCCTCAGCTACC
AAACCCACCAATGTACCCAGTGCAGCCACTGCCTCCACTGATCCCAGATAGACCTCTT
GAGTCATGGCCAGTACCTGATAA

AME_Hap_3
GGGGGAGATGTGAAATCGGTGTGCATGATGCACCAAAGTCCCTTTCAAGCTCTCCATC
CAATGCCACCACCTCTCCACCAGGTACAGCAACAGCCACCTCTGAACCCACACATGCA
GCTACCTGGACATAACACATTTGTGCCAATGACTGGACAGAATACATTAGTGCCACAGT
ATCAACCAGCTCACACAGGTCCAGTTCACCAGCCAGTTCCACCAGTTGCAGGAGAGC
CACCAATGCACCCTCAGCCACCAGCACATCCAAATCAGCCAATGCACCCTCAGCTACC
AAACCCACCAATGTACCCAGTGCAGCCACTGCCTCCACTGATCCCAGATAGACCTCTT
GAGTCATGGCCAGTACCTGATAA

AME_Hap_4
GGGGGAGATGTGAAATCGGTGTGCATGATGCACCAAAGTCCCTTTCAAGCTCTCCATC
CAATGCCACCACCTCTCCACCAGGTACAGCAACAGCCACCTCTGAACCCACACATGCA
GCTACCTGGACATAACACATTTGTGCCAATGACTGGACAGAATACATTAGTGCCACAGT
ATCAACCAGCTCACGCAGGTCCAGTTCACCAGCCAGTTCCACCAGTTGCAGGAGAGC
CTACAATGCACCCTCAGCCACCAGCACATCCAAATCAGCCAATGCACCCTCAGCTACC
AAACCCACCAATGTACCCAGTGCAGCCACTGCCTCCACTGATCCCAGATAGACCTCTT
GAGTCATGGCCAGTACCTGATAA

AME_Hap_5
GGGGGAGATGTGAAATCGGTGTGCATGATGCACCAAAGTCCCTTTCAAGCTCTCCATC
C---GCCACCACCTCTCCACCAGGTACAGCAACAGCCACCTCTGAACCCACACATGCAG
CTACCTGGACATAACACATTTGTGCCAATGACTGGACAGAATACATTAGTGCCACAGTA
TCAACCAGCTCACGCAGGTCCAGTTCACCAGCCAGTTCCACCAGTTGCAGGAGAGCCT

ACAATGCACCCTCAGCCACCAGCACATCCAAATCAGCCAATGCACCCTCAGCTACCAA
ACCCACCAATGTACCCAGTGCAGCCACTGCCTCCACTGATCCCAGATAGACCTCTTGA
GTCATGGCCAGTACCTGATAA

AME_Hap_6
GGGGGAGATGTGAAATCGGTGTGCATGATGCACCAAAGTCCCTTTCAAGCTCTCCATC
CAATGCCACCACCTCTCCACCAGGTACAGCAACAGCTACCTCTGAACCCACACATGCA
GCTACCTGGACATAACACATTTGTGCCAATGACTGGACAGAATACATTAGTGCCACAGT
ATCAACCAGCTCACGCAGGTCCAGTTCACCAGCCAGTTCCACCAGTTGCAGGAGAGC
CATCAATGCACCCTCAGCCACCAGCACATCCAAATCAGCCAATGCACCCTCAGCTACC
AAACCCACCAATGTACCCAGTGCAGCCACTGCCTCCACTGATCCCAGATAGACCTCTT
GAGTCATGGCCAGTACCTGATAA

AME_Hap_7
GGGGGAGATGTGAAATCGGTGTGCATGATGCACCAAAGTCCCTTTCAAGCTCTCCATC
CAATGCCACCACCTCTCCACCAGGTACAGCAACAGCCACCTCTGAACCCACACATGCA
GCTACCTGGACATAACACATTTGTGCCAATGACTGGACAGAATACATTAGTGCCACAGT
ATCCACCAGCTCACGCAGGTCCAGTTCACCAGCCAGTTGCACCAGTTGCAGGAGAGC
CACCAATGCACCCTCAGCCACCAGCACATCCAAATCAGCCAATGCACCCTCAGCTACC
AAACCCACCAATGTACCCAGTGCAGCCACTGCCTCCACTGATCCCAGATAGACCTCTT
GAGTCATGGCCAGTACCTGATAA

AME_Hap_8
GGGGGAGATGTGAAATCGGTGTGCATGATGCACCAAAGTCCCTTTCATGCTCTCCATCC
AATGCCACCACCTCTCCACCAGGTACAGCAACAGCCACCTCTGAACCCACACATGCAG
CTACCTGGACATAACACATTTGTGCCAATGACTGGACAGAATACATTAGTGCCACAGTA
TCAACCAGCTCACGCAGGTCCAGTTCACCAGCCAGTTCCACCAGTTGCAGGAGAGCC
ACCAATGCACCCTCAGCCACCAGCACATCCAAATCAGCCAATGCACCCTCAGCTACCA
AACCCACCAATGTACCCAGTGCAGCCACTGCCTCCACTGATCCCAGATAGACCTCTTG
AGTCATGGCCAGTACCTGATAA

The 15 RAG-1 genotype sequences (aligned) detected in *P. theobaldi*

RAG-1_Hap_1
TGACGATACACAAGCTCTTCTCAGGAAGAAGGAGAAAAGAGCTACGTCTTGGCCTGAT
CTCCTTGCCAAAGTGTTCAAGATTGATGTCAGAGGGGACGTTGACACAATCCACCCCA
CCAATTTTTGCCACAATTGCCGGAATGTGATTCAAAGAAAGTTCAGCAATTCCCCAGGT
GAAGTGTATTTTCCAAGGAAAGATGCCATGGAGTGGCAACCGCATTCGTTGAGCTGCG
ATGTCTGTGGCACTTCCTTCCGTGGGGTAAAGAGAAAGAAGCGGCCCTTAAATCCACC
GTTGAGCAAAAAGCCCAGGGTTGTAGCTGGAGGTGCTAGAAAACCAAGCTATGTGAG
GAATGTAAAACAAGTGAACAACAAGGCTCTAATGAAAAAGATTGCCAATTGCAAGAA
AATCCACCTTAGTACCAAGATCCTTGCAGTAGATTATCCTGTGGATTTCGTAAAGTCAGT
CTCCTGCCAGGTCTGTGAGCATATTCTGGCTGATCCTGTAGAAACCACATGCAAGCACT
TATTTTGCAGGGCCTGCCTCCTTAAATGCCTCAAAGTCATGGGAAGTTTTTGTCCAGTT
TGTTGCTATCCTTGTTTTCCGACAGATCTGGTGAGCCCTGTGAAATCCTTCCTGAACAT
CCTCAACAACCTGGCTCTGAGATGTCCCGTTAAAGATTGCCAAGAGGAGGTTGTTTTG
GGAAAATACAGCCACCACTTATCCAGCCACAAAGAGGTGGAAGACAAAGAGGGCTAC
ATGTATATAAACAAAGGTGGCCGGCCGAGACAGCATCTGCTTTCCCTGACCCGGAGAG
CTCAAAAACACCGTCTAAGAGATCTCAAACTTCAAGTGAAAGCTTTTGCTGAGAAAG
AAGAA

RAG-1_Hap_2
TGACGATACACAAGCTCTTCTCAGGAAGAAGGAGAAAAGAGCTACGTCTTGGCCTGAT
CTCCTTGCCAAAGTGTTCAAGATTGATGTCAGAGGGGACGTTGACACAATCCACCCCA
CCAATTTTTGCCACAATTGCCGGAATGTGATTCAAAGAAAGTTCAGCAATTCCCCAGGT
GAAGTGTATTTTCCAAGGAAAGATGCCATGGAGTGGCAACCGCATTCGTTGAGCTGCG
ATGTCTGTGGCACTTCCTTCCGTGGGGTAAAGAGAAAGAAGCGGCCCTTAAATCCACC
GTTGAGCAAAAAGCCCAGGGTTGTAGCTGGAGGTGCTAGAAAACCAAGCTATGTGAG
GAATGTAAAACAAGTGAACAACAAGGCTCTAATGAAAAAGATTGCCAATTGCAAGAA
AATCCACCTTAGTACCAAGATCCTTGCAGTAGATTATCCTGTGGATTTCGTAAAGTCAGT
CTCCTGCCAGGTCTGTGAGCATATTCTGGCTGATCCTGTAGAAACCACATGCAAGCACT
TATTTTGCAGGGCCTGCCTCCTTAAATGCCTCAAAGTCATGGGAAGTTTTTGTCCAGTT
TGTTGCTATCCTTGTTTTCCGACAGATCTGGTGAGCCCTGTGAAATCCTTCCTGAACAT
CCTCAACAACCTGGCTCTGAGATGTCCCGTTAAAGATTGCCAAGAGGAGGTTGTTTTG
GGAAAATACAGTCACCACTTATCCAGCCACAAAGAGGTGGAAGACAAAGAGGGCTAC
ATGTATATAAACAAAGGTGGCCGGCCGAGACAGCATCTGCTTTCCCTGACCCGGAGAG
CTCAAAAACACCGTCTAAGAGATCTCAAACTTCAAGTGAAAGCTTTTGCTGAGAAAG
AAGAA

RAG-1_Hap_3
TGACGATACACAAGCTCTTCTCAGGAAGAAGGAGAAAAGAGCTACGTCTTGGCCTGAT
CTCCTTGCCAAAGTGTTCAAGATTGATGTCAGAGGGGACGTTGACACAATCCACCCCA
CCAATTTTTGCCACAATTGCCGGAATGTGATTCAAAGAAAGTTCAGCAATTCCCCAGGT
GAAGTGTATTTTCCAAGGAAAGATGCCATGGAGTGGCAACCGCATTCGTTGAGCTGCG

ATGTCTGTGGCACTTCCTTCCGTGGGGTAAAGAGAAAGAAGCGGCCCTTAAATCCACC
GTTGAGCAAAAAGCCCAGGGTTGTAGCTGGAGGTGCTAGAAAACCAAGCTATGTGAG
GAATGTAAAACAAGTGAACAACAAGGCTCTAATGAAAAAGATTGCCAATTGCAAGAA
AATCCACCTTAGTACCAAGATCCTTGCAGTAGATTATCCTGTGGATTTCGTAAAGTCAGT
CTCCTGCCAGGTCTGTGAGCATATTCTGGCTGATCCTGTAGAAACCACATGCAAGCACT
TATTTTGCAGGGCCTGCCTCCTTAAATGCCTCAAAGTCATGGGAAGTTTTTGTCCAGTT
TGTTGCTATCCTTGTTTTCCGACAGATCTGGTGAGCCCTGTGAAATCCTTCCTGAACAT
CCTCAACAACCTGGCTCTGAGATGTCCCGTTAAAGATTGCCAAGAGGAGGTTGTTTTG
GGAAAATACAGCCACCACTTATCCAGCCACAAAGAGGTGGAAGACAAAGAGGGCTAC
ATGTATATAAACAAAGGTGGCCGGCCGAGACAGCATCTCCTTTCCCTGACCCGGAGAG
CTCAAAAACACCGTCTAAGAGATCTCAAACTTCAAGTGAAAGCTTTTGCTGAGAAAG
AAGAA

RAG-1_Hap_4
TGACGATACACAAGCTCTTCTCAGGAAGAAGGAGAAAAGAGCTACGTCTTGGCCTGAT
CTCCTTGCCAAAGTGTTCAAGATTGATGTCAGAGGGGACGTTGACACAATCCACCCCA
CCAATTTTTGCCACAATTGCCGGAATGTGATTCAAAGAAAGTTCAGCAATTCCCCAGGT
GAAGTGTATTTTCCAAGGAAAGATTCCATGGAGTGGCAACCGCATTCGTTGAGCTGCG
ATGTCTGTGGCACTTCCTTCCGTGGGGTAAAGAGAAAGAAGCGGCCCTTAAATCCACC
GTTGAGCAAAAAGCCCAGGGTTGTAGCTGGAGGTGCTAGAAAACCAAGCTATGTGAG
GAATGTAAAACAAGTGAACAACAAGGCTCTAATGAAAAAGATTGCCAATTGCAAGAA
AATCCACCTTAGTACCAAGATCCTTGCAGTAGATTATCCTGTGGATTTCGTAAAGTCAGT
CTCCTGCCAGGTCTGTGAGCATATTCTGGCTGATCCTGTAGAAACCACATGCAAGCACT
TATTTTGCAGGGCCTGCCTCCTTAAATGCCTCAAAGTCATGGGAAGTTTTTGTCCAGTT
TGTTGCTATCCTTGTTTTCCGACAGATCTGGTGAGCCCTGTGAAATCCTTCCTGAACAT
CCTCAACAACCTGGCTCTGAGATGTCCCGTTAAAGATTGCCAAGAGGAGGTTGTTTTG
GGAAAATACAGCCACCACTTATCCAGCCACAAAGAGGTGGAAGACAAAGAGGGCTAC
ATGTATATAAACAAAGGTGGCCGGCCGAGACAGCATCTGCTTTCCCTGACCCGGAGAG
CTCAAAAACACCGTCTAAGAGATCTCAAACTTCAAGTGAAAGCTTTTGCTGAGAAAG
AAGAA

RAG-1_Hap_5
TGATGATACACAAGCTCTTCTCAGGAAGAAGGAGAAAAGAGCTACGTCTTGGCCTGAT
CTCCTTGCCAAAGTGTTCAAGATTGATGTCAGAGGGGACATTGACACAATCCACCCCA
CCAATTTTTGCCACAATTGCCGGAATGTGATTCAAAGAAAGTTCAGCAATTCCCCAGGT
GAAGTGTATTTTCCAAGGAAAGATGCCATGGAGTGGCAACCGCATTCATTGAGCTGCG
ATGTCTGTGGCACTTCCTTCCGTGGGGTAAAGAGAAAGAAGCGGCCCTTAAATCCACC
GTTGAGCAAAAAGCCCAGGGTTGTAGCTGGAGGTGCTAGAAAACCAAGCTATGTGAG
GAATGTAAAACAAGTGAACAACAAGGCTCTAATGAAAAAGATTGCCAATTGCAAGAA
AATCCACCTTAGTACCAAGATCCTTGCAGTAGATTATCCTGTGGATTTCGTAAAGTCAGT
CTCCTGCCAGGTCTGTGAGCATATTCTGGCTGATCCTGTAGAAACCACATGCAAGCACT
TATTTTGCAGGGCCTGCCTCCTTAAATGCCTCAAAGTCATGGGAAGTTTTTGTCCAGTT
TGTTGCTATCCTTGTTTTCCGACAGATCTGGTGAGCCCTGTGAAATCCTTCCTGAACAT
CCTCAACAACCTGGCTCTGAGATGTCCCGTTAAAGATTGCCAAGAGGAGGTTGTTTTG

GGAAAATACAGCCACCACTTATCCAGCCACAAAGAGGTGGAAGACAAAGAGGGCTAC
ATGTATATAAACAAAGGTGGCCGGCCGAGACAGCATCTGCTTTCCCTGACCCGGAGAG
CTCAAAAACACCGTCTAAGAGATCTCAAACTTCAAGTGAAAGCTTTTGCTGAGAAAG
AAGAA

RAG-1_Hap_6
TGACGATACACAAGCTCTTCTCAGGAAGAAGGAGAAAAGAGCCACGTCTTGGCCTGA
TCTCCTTGCCAAAGTGTTCAAGATTGATGTCAGAGGGGACGTTGACACAATCCACCCC
ACCAATTTTTGCCACAATTGCCGGAATGTGATTCAAAGAAAGTTCAGCAATTCCCCAG
GTGAAGTGTATTTTCCAAGGAAAGATGCCATGGAGTGGCAACCGCATTCGTTGAGCTG
CGATGTCTGTGGCACTTCCTTCCGTGGGGTAAAGAGAAAGAAGCGGCCCTTAAATCCA
CCGTTGAGCAAAAAGCCCAGGGTTGTAGGTGGAGGTGCTAGAAAACCAAGCTATGTG
AGGAATGTAAAACAAGTGAACAACAAGGCTCTAATGAAAAAGATTGCCAATTGCAAG
AAAATCCACCTTAGTACCAAGATCCTTGCAGTAGATTATCCTGTGGATTTCGTAAAGTC
AGTCTCCTGCCAGGTCTGTGAGCATATTCTGGCTGATCCTGTAGAAACCACATGCAAGC
ACTTATTTTGCAGGGCCTGCCTCCTTAAATGCCTCAAAGTCATGGGAAGTTTTTGTCCA
GTTTGTTGCTATCCTTGTTTTCCGACAGATCTGGTGAGCCCTGTGAAATCCTTCCTGAA
CATCCTCAACAACCTGGCTCTGAGATGTCCCGTTAAAGATTGCCAAGAGGAGGTTGTT
TTGGGAAAATACAGCCACCACTTATCCAGCCACAAAGAGGTGGAAGACAAAGAGGGC
TACATGTATATAAACAAAGGTGGCCGGCCGAGACAGCATCTGCTTTCCCTGACCCGGA
GAGCTCAAAAACACCGTCTAAGAGATCTCAAACTTCAAGTGAAAGCTTTTGCTGAGA
AAGAAGAA

RAG-1_Hap_7
TGACGATACACAAGCTCTTCTCAGGAAGAAGGAGAAAAGAGCCACGTCTTGGCCTGA
TCTCCTTGCCAAAGTGTTCAAGATTGATGTCAGAGGGGACGTTGACACAATCCACCCC
ACCAATTTTTGCCACAATTGCCGGAATGTGATTCAAAGAAAGTTCAGCAATTCCCCAG
GTGAAGTGTATTTTCCAAGGAAAGATGCCATGGAGTGGCAACCGCATTCGTTGAGCTG
CGATGTCTGTGGCACTTCCTTCCGTGGGGTAAAGAGAAAGAAGCGGCCCTTAAATCCA
CCGTTGAGCAAAAAGCCCAGGGTTGTAGGTGGAGGTGCTAGAAAACCAAGCTATGTG
AGGAATGTAAAACAAGTGAACAACAAGGCTCTAATGAAAAAGATTGCCAATTGCAAG
AAAATCCACCTTAGTACCAAGATCCTTGCAATAGATTATCCTGTGGATTTCGTAAAGTCA
GTCTCCTGCCAGGTCTGTGAGCATATTCTGGCTGATCCTGTAGAAACCACATGCAAGCA
CTTATTTTGCAGGGCCTGCCTCCTTAAATGCCTCAAAGTCATGGGAAGTTTTTGTCCAG
TTTGTTGCTATCCTTGTTTTCTGACAGATCTGGTGAGCCCTGTGAAATCCTTCCTGAACA
TCCTCAACAACCTGGCTCTGAGATGTCCCGTTAAAGATTGCCAAGAGGAGGTTGTTTT
GGGAAAATACAGCCACCACTTATCCAGCCACAAAGAGGTGGAAGACAAAGAGGGCTA
CATGTATATAAACAAAGGTGGCCGGCCGAGACAGCATCTGCTTTCCCTGACCCGGAGA
GCTCAAAAACACCGTCTAAGAGATCTCAAACTTCAAGTGAAAGCTTTTGCTGAGAAA
GAAGAA

RAG-1_Hap_8
TGACGATACACAAGCTCTTCTCAGGAAGAAGGAGAAAAGAGCTACGTCTTGGCCTGAT
CTCCTTGCCAAAGTGTTCAAGATTGATGTCAGAGGGGACGTTGACACAATCCACCCCA

CCAATTTTTGCCACAATTGCCGGAATGTGATTCAAAGAAAGTTCAGCAATTCCCCAGGT
GAAGTGTATTTTCCAAGGAAAGATGCCATGGAGTGGCAACCGCATTCATTGAGCTGCG
ATGTCTGTGGCACTTCCTTCCGTGGGGTAAAGAGAAAGAAGCGGCCCTTAAATCCACC
GTTGAGCAAAAAGCCCAGGGTTGTAGCTGGAGGTGCTAGAAAACCAAGCTATGTGAG
GAATGTAAAACAAGTGAACAACAAGGCTCTAATGAAAAAGATTGCCAATTGCAAGAA
AATCCACCTTAGTACCAAGATCCTTGCAGTAGATTATCCTGTGGATTTCGTAAAGTCAGT
CTCCTGCCAGGTCTGTGAGCATATTCTGGCTGATCCTGTAGAAACCACATGCAAGCACT
TATTTTGCAGGGCCTGCCTCCTTAAATGCCTCAAAGTCATGGGAAGTTTTTGTCCAGTT
TGTTGCTATCCTTGTTTTCCGACAGATCTGGTGAGCCCTGTGAAATCCTTCCTGAACAT
CCTCAACAACCTGGCTCTGAGATGTCCCGTTAAAGATTGCCAAGAGGAGGTTGTTTTG
GGAAAATACAGCCACCACTTATCCAGCCACAAAGAGGTGGAAGACAAAGAGGGCTAC
ATGTATATAAACAAAGGTGGCCGGCCGAGACAGCATCTGCTTTCCCTGACCCGGAGAG
CTCAAAAACACCGTCTAAGAGATCTCAAACTTCAAGTGAAAGCTTTTGCTGAGAAAG
AAGAA

RAG-1_Hap_9
TGACGATACACAAGCTCTTCTCAGGAAGAAGGAGAAAAGAGCTACGTCTTGGCCTGAT
CTCCTTGCCAAAGTGTTCAAGATTGATGTCAGAGGGGACGTTGACACAATCCACCCCA
CCAATTTTTGCCACAATTGCCGGAATGTGATTCAAAGAAAGTTCAGCAATTCCCCAGGT
GAAGTGTATTTTCCAAGGAAAGATGCCATGGAGTGGCAACCGCATTCGTTGAGCTGCG
ATGTCTGTGGCACTTCCTTCCGTGGGGTAAAGAGAAAGAAGCGGCCCTTAAATCCACC
GTTGAGCAAAAAGCCCAGGGTTGTAGGTGGAGGTGCTAGAAAACCAAGCTATGTGAG
GAATGTAAAACAAGTGAACAACAAGGCTCTAATGAAAAAGATTGCCAATTGCAAGAA
AATCCACCTTAGTACCAAGATCCTTGCAGTAGATTATCCTGTGGATTTCGTAAAGTCAGT
CTCCTGCCAGGTCTGTGAGCATATTCTGGCTGATCCTGTAGAAACCACATGCAAGCACT
TATTTTGCAGGGCCTGCCTCCTTAAATGCCTCAAAGTCATGGGAAGTTTTTGTCCAGTT
TGTTGCTATCCTTGTTTTCCGACAGATCTGGTGAGCCCTGTGAAATCCTTCCTGAACAT
CCTCAACAACCTGGCTCTGAGATGTCCCGTTAAAGATTGCCAAGAGGAGGTTGTTTTG
GGAAAATACAGCCACCACTTATCCAGCCACAAAGAGGTGGAAGACAAAGAGGGCTAC
ATGTATATAAACAAAGGTGGCCGGCCGAGACAGCATCTGCTTTCCCTGACCCGGAGAG
CTCAAAAACACCGTCTAAGAGATCTCAAACTTCAAGTGAAAGCTTTTGCTGAGAAAG
AAGAA

RAG-1_Hap_10
TGATGATACACAAGCTCTTCTCAGGAAGAAGGAGAAAAGAGCTACGTCTTGGCCTGAT
CTCCTTGCCAAAGTGTTCAAGATTGATGTCAGAGGGGACGTTGACACAATCCACCCCA
CCAATTTTTGCCACAATTGCCGGAATGTGATTCAAAGAAAGTTCAGCAATTCCCCAGGT
GAAGTGTATTTTCCAAGGAAAGATGCCATGGAGTGGCAACCGCATTCATTGAGCTGCG
ATGTCTGTGGCACTTCCTTCCGTGGGGTAAAGAGAAAGAAGCGGCCCTTAAATCCACC
GTTGAGCAAAAAGCCCAGGGTTGTAGCTGGAGGTGCTAGAAAACCAAGCTATGTGAG
GAATGTAAAACAAGTGAACAACAAGGCTCTAATGAAAAAGATTGCCAATTGCAAGAA
AATCCACCTTAGTACCAAGATCCTTGCAGTAGATTATCCTGTGGATTTCGTAAAGTCAGT
CTCCTGCCAGGTCTGTGAGCATATTCTGGCTGATCCTGTAGAAACCACATGCAAGCACT
TATTTTGCAGGGCCTGCCTCCTTAAATGCCTCAAAGTCATGGGAAGTTTTTGTCCAGTT

TGTTGCTATCCTTGTTTTCCGACAGATCTGGTGAGCCCTGTGAAATCCTTCCTGAACAT
CCTCAACAACCTGGCTCTGAGATGTCCCGTTAAAGATTGCCAAGAGGAGGTTGTTTTG
GGAAAATACAGCCACCACTTATCCAGCCACAAAGAGGTGGAAGACAAAGAGGGCTAC
ATGTATATAAACAAAGGTGGCCGGCCGAGACAGCATCTGCTTTCCCTGACCCGGAGAG
CTCAAAAACACCGTCTAAGAGATCTCAAACTTCAAGTGAAAGCTTTTGCTGAGAAAG
AAGAA

RAG-1_Hap_11
TGATGATACACAAGCTCTTCTCAGGAAGAAGGAGAAAAGAGCTACGTCTTGGCCTGAT
CTCCTTGCCAAAGTGTTCAAGATTGATGTCAGAGGGGACGTTGACACAATCCACCCCA
CCAATTTTTGCCACAATTGCCGGAATGTGATTCAAAGAAAGTTCAGCAATTCCCCAGGT
GAAGTGTATTTTCCAAGGAAAGATGCCATGGAGTGGCAACCGCATTCATTGAGCTGCG
ATGTCTGTGGCACTTCCTTCCGTGGGGTAAAGAGAAAGAGGCGGCCCTTAAATCCACC
GTTGAGCAAAAAGCCCAGGGTTGTAGCTGGAGGTGCTAGAAAACCAAGCTATGTGAG
GAATGTAAAACAAGTGAACAACAAGGCTCTAATGAAAAAGATTGCCAATTGCAAGAA
AATCCACCTTAGTACCAAGATCCTTGCAGTAGATTATCCTGTGGATTTCGTAAAGTCAGT
CTCCTGCCAGGTCTGTGAGCATATTCTGGCTGATCCTGTAGAAACCACATGCAAGCACT
TATTTTGCAGGGCCTGCCTCCTTAAATGCCTCAAAGTCATGGGAAGTTTTTGTCCAGTT
TGTTGCTATCCTTGTTTTCCGACAGATCTGGTGAGCCCTGTGAAATCCTTCCTGAACAT
CCTCAACAACCTGGCTCTGAGATGTCCCGTTAAAGATTGCCAAGAGGAGGTTGTTTTG
GGAAAATACAGCCACCACTTATCCAGCCACAAAGAGGTGGAAGACAAAGAGGGCTAC
ATGTATATAAACAAAGGTGGCCGGCCGAGACAGCATCTGCTTTCCCTGACCCGGAGAG
CTCAAAAACACCGTCTAAGAGATCTCAAACTTCAAGTGAAAGCTTTTGCTGAGAAAG
AAGAA

RAG-1_Hap_12
TGATGATACACAAGCTCTTCTCAGGAAGAAGGAGAAAAGAGCTACGTCTTGGCCTGAT
CTCCTTGCCAAAGTGTTCAAGATTGATGTCAGAGGGGACGTTGACACAATCCACCCCA
CCAATTTTTGCCACAATTGCCGGAATGTGATTCAAAGAAAGTTCAGCAATTCCCCAGGT
GAAGTGTATTTTCCAAGGAAAGATGCCATGGAGTGGCAACCGCATTCATTGAGCTGCG
ATGTCTGTGGCACTTCCTTCCGTGGGGTAAAGAGAAAGAGGCGGCCCTTAAATCCACC
ATTGAGCAAAAAGCCCAGGGTTGTAGCTGGAGGTGCTAGAAAACCAAGCTATGTGAG
GAATGTAAAACAAGTGAACAACAAGGCTCTAATGAAAAAGATTGCCAATTGCAAGAA
AATCCACCTTAGTACCAAGATCCTTGCAGTAGATTATCCTGTGGATTTCGTAAAGTCAGT
CTCCTGCCAGGTCTGTGAGCATATTCTGGCTGATCCTGTAGAAACCACATGCAAGCACT
TATTTTGCAGGGCCTGCCTCCTTAAATGCCTCAAAGTCATGGGAAGTTTTTGTCCAGTT
TGTTGCTATCCTTGTTTTCCGACAGATCTGGTGAGCCCTGTGAAATCCTTCCTGAACAT
CCTCAACAACCTGGCTCTGAGATGTCCCGTTAAAGATTGCCAAGAGGAGGTTGTTTTG
GGAAAATACAGCCACCACTTATCCAGCCACAAAGAGGTGGAAGACAAAGAGGGCTAC
ATGTATATAAACAAAGGTGGCCGGCCGAGACAGCATCTGCTTTCCCTGACCCGGAGAG
CTCAAAAACACCGTCTAAGAGATCTCAAACTTCAAGTGAAAGCTTTTGCTGAGAAAG
AAGAA

RAG-1_Hap_13

TGATGATACACAAGCTCTTCTCAGGAAGAAGGAGAAAAGAGCTACGTCTTGGCCTGAT
CTCCTTGCCAAAGTGTTCAAGATTGATGTCAGAGGGGACGTTGACACAATCCACCCCA
CCAATTTTTGCCACAATTGCCGGAATGTGATTCAAAGAAAGTTCAGCAATTCCCCAGGT
GAAGTGTATTTTCCAAGGAAAGATGCCATGGAGTGGCAACCGCATTCATTGAGCTGCG
ATGTCTGTGGCACTTCCTTCCGTGGGGTAAAGAGAAAGAGGCGGCCCTTAAATCCACC
GTTGAGCAAAAAGCCCAGGGTTGTAGCTGGAGGTGCTAGAAAACCAAGCTATGTGAG
GAATGTAAAACAAGTGAACAACAAGGCTCTAATGAAAAAGATTGCCAATTGCAAGAA
AATCCACCTTAGTACCAAGATCCTTGCAGTAGATTATCCTGTGGATTTCGTAAAGTCAGT
CTCCTGCCAGGTCTGTGAGCATATTCTGGCTGATCCTGTAGAAACCACATGCAAGCACT
TATTTTGCAGGGCCTGCCTCCTTAAATGCCTCAAAGTCATGGGAAGTTTTTGTCCAGTT
TGTTGCTATCCTTGTTTTCCGACAGATCTGGTGAGCCCTGTGAAATCCTTCCTGAACAT
CCTCAACAACCTGGCTCTGAGATGTCCCGTTAAAGATTGCCAAGAAGAGGTTGTTTTG
GGAAAATACAGCCACCACTTATCCAGCCACAAAGAGGTGGAAGACAAAGAGGGCTAC
ATGTATATAAACAAAGGTGGCCGGCCGAGACAGCATCTGCTTTCCCTGACCCGGAGAG
CTCAAAAACACCGTCTAAGAGATCTCAAACTTCAAGTGAAAGCTTTTGCTGAGAAAG
AAGAA

RAG-1_Hap_14
TGATGATACACAAGCTCTTCTCAGGAAGAAGGAGAAAAGAGCTACGTCTTGGCCTGAT
CTCCTTGCCAAAGTGTTCAAGATTGATGTCAGAGGGGACGTTGACACAATCCACCCCA
CCAATTTTTGCCACAATTGCCGGAATGTGATTCAAAGAAAGTTCAGCAATTCCCCAGGT
GAAGTGTATTTTCCAAGGAAAGATGCCATGGAGTGGCAACCGCATTCATTGAGCTGCG
ATGTCTGTGGCACTTCCTTCCGTGGGGTAAAGAGAAAGAAGCGGCCCTTAAATCCACC
GTTGAGCAAAAAGCCCAGGGTTGTAGCTGGAGGTGCTAGAAAACCAAGCTATGTGAG
GAATGTAAAACAAGTGAACAACAAGGCTCTAATGAAAAAGATTGCCAATTGCAAGAA
AATCCACCTTAGTACCAAGATCCTTGCAGTAGATTATCCTGTGGATTTCGTAAAGTCAGT
CTCCTGCCAGGTCTGTGAGCATATTCTGGCTGATCCTGTAGAAACCACATGCAAGCACT
TATTTTGCAGGGCCTGCCTCCTTAAATGCCTCAAAGTCATGGGAAGTTTTTGTCCAGTT
TGTTGCTATCCTTGTTTTCCAACAGATCTGGTGAGCCCTGTGAAATCCTTCCTGAACAT
CCTCAACAACCTGGCTCTGAGATGTCCCGTTAAAGATTGCCAAGAGGAGGTTGTTTTG
GGAAAATACAGCCACCACTTATCCAGCCACAAAGAGGTGGAAGACAAAGAGGGCTAC
ATGTATATAAACAAAGGTGGCCGGCCGAGACAGCATCTGCTTTCCCTGACCCGGAGAG
CTCAAAAACACCGTCTAAGAGATCTCAAACTTCAAGTGAAAGCTTTTGCTGAGAAAG
AAGAA

RAG-1_Hap_15
TGATGATACACAAGCTCTTCTCAGGAAGAAGGAGAAAAGAGCTACGTCTTGGCCTGAT
CTCCTTGCCAAAGTGTTCAAGATTGATGTCAGAGGGGACGTTGACACAATCCACCCCA
CCAATTTTTGCCACAATTGCCGGAATGTGATTCAAAGAAAGTTCAGCAATTCCCCAGGT
GAAGTGTATTTTCCAAGGAAAGATGCCATGGAGTGGCAACCGCATTCGTTGAGCTGCG
ATGTCTGTGGCACTTCCTTCCGTGGGGTAAAGAGAAAGAAGCGGCCCTTAAATCCACC
GTTGAGCAAAAAGCCCAGGGTTGTAGCTGGAGGTGCTAGAAAACCAAGCTATGTGAG
GAATGTAAAACAAGTGAACAACAAGGCTCTAATGAAAAAGATTGCCAATTGCAAGAA
AATCCACCTTAGTACCAAGATCCTTGCAGTAGATTATCCTGTGGATTTCGTAAAGTCAGT

CTCCTGCCAGGTCTGTGAGCATATTCTGGCTGATCCTGTAGAAACCACATGCAAGCACT
TATTTTGCAGGGCCTGCCTCCTTAAATGCCTCAAAGTCATGGGAAGTTTTTGTCCAGTT
TGTTGCTATCCTTGTTTTCCGACAGATCTGGTGAGCCCTGTGAAATCCTTCCTGAACAT
CCTCAACAACCTGGCTCTGAGATGTCCCGTTAAAGATTGCCAAGAGGAGGTTGTTTTG
GGAAAATACAGCCACCACTTATCCAGCCACAAAGAGGTGGAAGACAAAGAGGGCTAC
ATGTATATAAACAAAGGTGGCCGGCCGAGACAGCATCTGCTTTCCCTGACCCGGAGAG
CTCAAAAACACCGTCTAAGAGATCTCAAACTTCAAGTGAAAGCTTTTGCTGAGAAAG
AAGAA

Supplementary Information S3

BPEC parsimony network. The pie charts for each mtDNA haplotype represent posterior probabilities for assignment to each of 6 possible clusters. Haplotypes were only assigned to four clusters with high posterior probabilities. These are indicated on the network as A1, A2-A4, B and S, and correspond to the labels used in Figure 3.

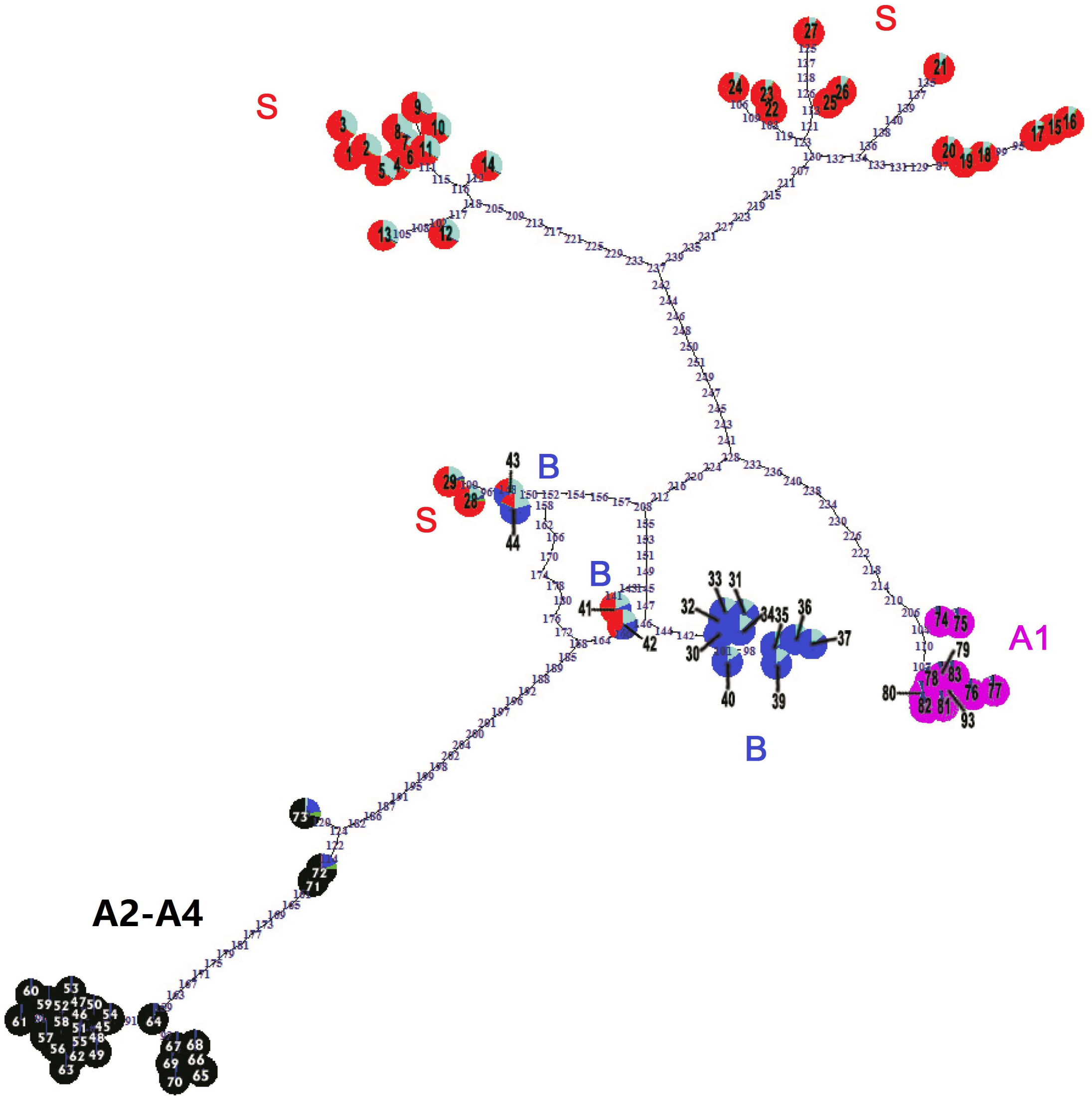

Supplementary Information S4

Median-joining networks for the two nuclear loci (AME and RAG-1).

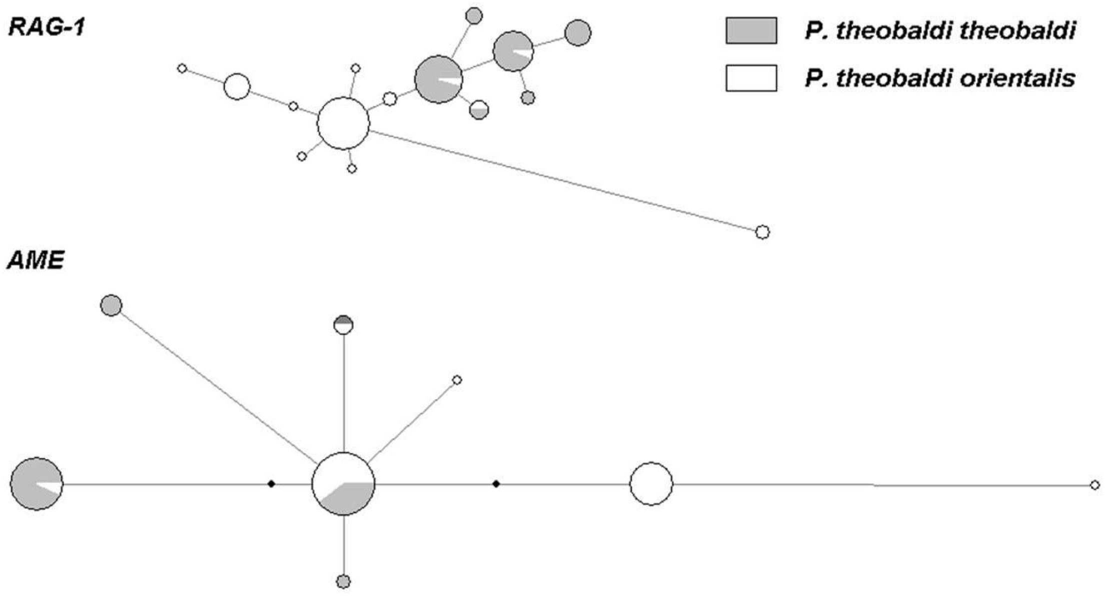

Supplementary Information S5

Additional detail on fine-scale mtDNA relationships within species other than *P. theobaldi* (see Figure 2a).

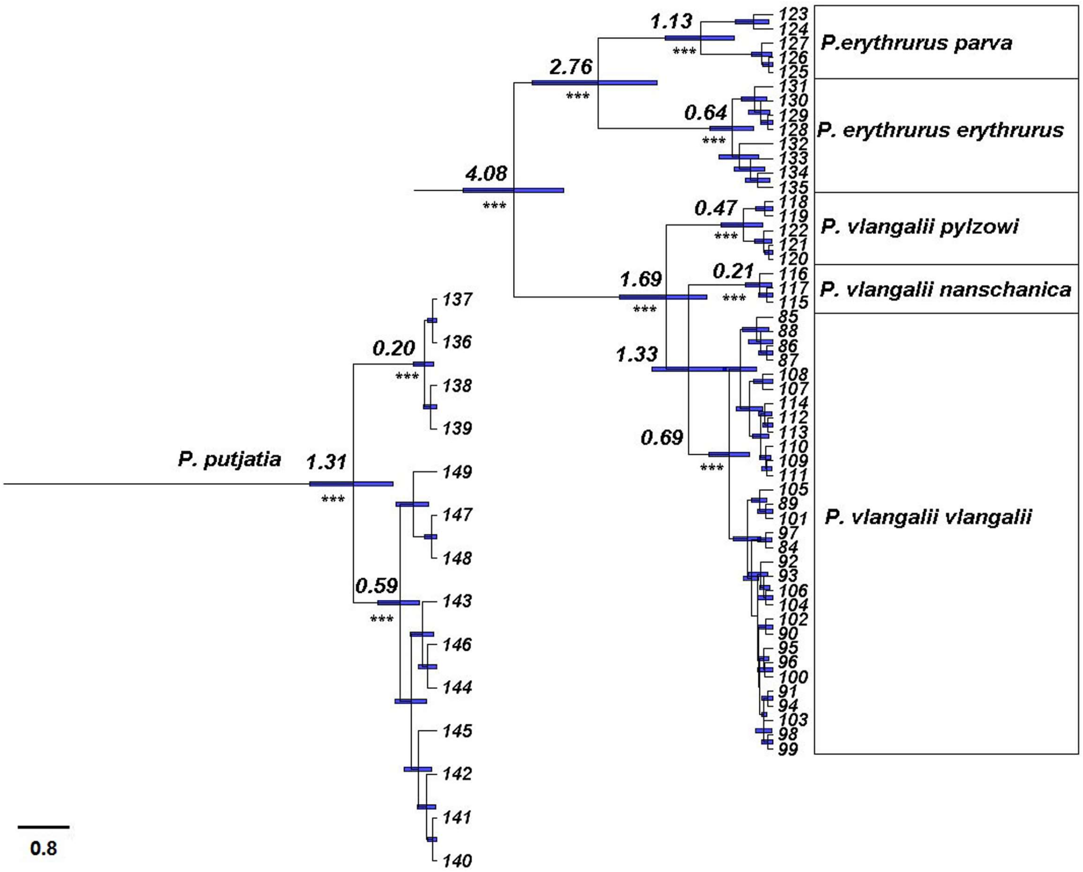

Supplement: Supplementary file 1 — The geography and timing of genetic divergence in the lizard Phrynocephalus theobaldi on the Qinghai-Tibetan plateau [file 41598_2017_2674_MOESM1_ESM.pdf]
